# PINK1 modulates Prdx2 to reduce lipotoxicity‐induced apoptosis and attenuate cardiac dysfunction in heart failure mice with a preserved ejection fraction

**DOI:** 10.1002/ctm2.70166

**Published:** 2025-01-06

**Authors:** Hao Zhang, Tianyu Xu, Xiyuan Mei, Qiming Zhao, Qiling Yang, Xianghui Zeng, Zhuang Ma, Haobin Zhou, Qingchun Zeng, Dingli Xu, Hao Ren

**Affiliations:** ^1^ State Key Laboratory of Organ Failure Research Department of Cardiology Nanfang Hospital Southern Medical University Guangzhou China; ^2^ Key Laboratory For Organ Failure Research Ministry of Education of the People's Republic of China Guangzhou China; ^3^ NHC Key Laboratory of Assisted Circulation, Department of Cardiology The First Affiliated Hospital of Sun Yat‐sen University Guangzhou China; ^4^ Department of Rheumatology Nanfang Hospital Southern Medical University Guangzhou China

## Abstract

**Introduction:**

Heart failure with preserved ejection fraction (HFpEF) is a complex condition characterized by metabolic dysfunction and myocardial lipotoxicity. The roles of PTEN‐induced kinase 1 (PINK1) and peroxiredoxin‐2 (Prdx2) in HFpEF pathogenesis remain unclear.

**Objective:**

This study aimed to investigate the interaction between PINK1 and Prdx2 to mitigate cardiac diastolic dysfunction in HFpEF.

**Methods:**

In vivo, PINK1‐knockout mice and cardiac‐specific PINK1‐overexpressing transgenic mice were used to establish an HFpEF mouse model via a high‐fat diet and L‐NAME. Myocardial lipotoxicity was induced by palmitic acid in vitro. Immunoprecipitation, western blotting and immunofluorescence analysis were performed to elucidate the molecular mechanisms involved.

**Results:**

We determined that PINK1 and Prdx2 were downregulated in the HFpEF mouse model. In vivo, PINK1 ablation exacerbated the reduction in Prdx2 expression, worsening cardiac dysfunction in HFpEF mice. Conversely, PINK1 overexpression restored Prdx2 levels and decreased reactive oxygen species and apoptosis, thereby reducing fibrosis and inflammation and ameliorating cardiac diastolic dysfunction in HFpEF mice. In vitro, an interaction between the N‐terminal region (amino acids 1–133) of PINK1 and Prdx2 was identified. The overexpression of PINK1 induced Prdx2 expression and effectively attenuated palmitic acid‐induced apoptosis through the c‐Jun amino‐terminal kinase (JNK) and mitogen‐activated protein kinase (p38) pathways, whereas siRNA‐mediated Prdx2 knockdown abolished the protective effect of PINK1.

**Conclusion:**

PINK1 alleviates lipotoxicity‐induced myocardial apoptosis and improves diastolic dysfunction in HFpEF through Prdx2, highlighting PINK1 overexpression as a potential therapeutic strategy for HFpEF.

**Key points:**

- Our investigation discloses a pivotal relationship between PINK1 and Prdx2 in the context of HFpEF.
- Notably, PINK1, in addition to its role in mitochondrial autophagy, can increase Prdx2 expression, effectively remove ROS and attenuate cardiomyocyte apoptosis by modulating the JNK and p38 pathways, thereby alleviating myocardial lipotoxicity and improving HFpEF cardiac function.
- Our studies offer valuable insights, opening avenues for the development of innovative therapeutic strategies in the prevention and treatment of HFpEF.

## Graphical abstract

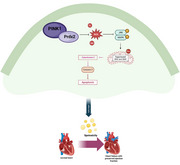

## 1 INTRODUCTION

Heart failure with preserved ejection fraction (HFpEF) represents an escalating public health challenge that constitutes approximately half of all heart failure (HF) cases.^1^, ^2^ Intermediate metabolic changes in the cardiometabolic system, which is a vital form of HFpEF, impact numerous signalling pathways in the heart. Long‐chain fatty acids (LCFAs) serve as crucial fuel for the heart; however, their excess can be detrimental, leading to the toxic accumulation of lipid by‐products, known as lipotoxicity.^3^, ^4^

Lipotoxicity is an important physiological process in HFpEF.^5^, ^6^ An imbalance in fatty acid oxidation and absorption can activate apoptotic signalling pathways, such as the c‐Jun amino‐terminal kinase (JNK) and mitogen‐activated protein kinase (p38) pathways,^7^, ^8^ enhance myocardial stiffness and contribute to the pathogenesis of diastolic dysfunction.^9^, ^10^, ^11^ Therefore, an effective therapeutic strategy to treat lipotoxicity may protect against HFpEF.

PTEN‐induced kinase 1 (PINK1) is vital for cardiomyocyte energy metabolism.^12^, ^13^, ^14^ Notably, mutations in PINK1, which result in impaired mitochondrial metabolism, are linked to myocardial injury.^15^ Despite the pivotal functions of PINK1 in mitophagy, previous studies revealed that PINK1 interacts with a variety of proteins and exerts multiple effects.^16^, ^17^ Recent studies suggest that PINK1 may be associated with the peroxiredoxin (Prdx) family.^18^

Among the six peroxiredoxin isoforms, peroxiredoxin‐2 (Prdx2) is primarily cytosolic and acts as an antioxidant, scavenging reactive oxygen species (ROS) such as hydrogen peroxide (H_2_O_2_) and organic hydroperoxides.^19^ Given their distinct intracellular distributions, these peroxides actively contribute to redox signal transduction (e.g., JNK and p38)^20^, ^21^, ^22^ to ultimately promote apoptosis.

This study investigates the role of PINK1 and Prdx2 in alleviating cardiac diastolic dysfunction in HFpEF. In vitro, neonatal rat cardiomyocytes (NRCMs) treated with palmitic acid demonstrated the effect of PINK1 on myocardial lipotoxicity, whereas the H9C2 cardiomyoblast cell line (H9C2) was utilized to examine the interaction between PINK1 and Prdx2. In vivo, PINK1‐knockout and cardiac‐specific PINK1‐overexpressing mice were subjected to a high‐fat diet and L‐NAME for 15 weeks to induce HFpEF. Cardiac function was assessed by echocardiography, with histological and molecular analyses to evaluate tissue structure and protein expression.

## 2 METHODS

### 2.1 Animals

WT C57BL/6 male mice (8–10 weeks old) were sourced from the Southern Medical University Animal Centre, while PINK1‐knockout mice (stock no. 017946) came from the Jackson Laboratory. A cardiac‐specific PINK1‐overexpressing mouse model was established by Shanghai Biomodel Organism Science & Technology Development.^16^ All animals were housed under pathogen‐free conditions. At the end of the study, all the animals were anaesthetized via isoflurane inhalation (4%) and then euthanized by cervical dislocation. All experimental protocols were approved by the Southern Medical University Institutional Animal Care and Use Committee (No. K2019022) and adhered to the NIH Guide for the Care and Use of Laboratory Animals.

### 2.2 Cell culture and treatments

NRCMs were isolated from 1‐ to 3‐day‐old SD rats following previously established methods. The cells were cultured in Dulbecco's modified Eagle's medium (DMEM) with 10% foetal bovine serum (FBS) in a humidified incubator at 37°C with 5% CO_2_. NRCMs were further transfected with plasmid or adenovirus (OBiO) according to the experimental design. PINK1 and Prdx2 siRNAs were purchased from Jiyuan Biotech (Guangzhou). Transfections of the siRNAs and negative control siRNAs were carried out via Lipofectamine 3000 in accordance with the manufacturer's guidelines. Following 48–72 h of transfection, NRCMs were exposed to palmitic acid (500 µmol/L) for 24 h. The generation and sequence information of the PINK1 (full length)‐GFP, PINK1 (1‐139aa)‐GFP and PINK1 (140‐580aa)‐GFP plasmids are presented in Supporting Information 3, and the H9C2 cell lineage was used in the structural domain experiments.

### 2.3 HFpEF model

The HFpEF model was constructed as described in a previous study.^23^ The mice were exposed to a combination of a HFD (60% calories from lard) and L‐NAME (.5 g/L in drinking water) for 15 weeks, while the control group was fed with standard (CHOW) diet for 15 weeks. The L‐NAME used in our experiments was purchased from Yuan Ye (S20013; Shanghai) and has a solubility of 100 mg/mL in water. The L‐NAME powder can be stored at −20°C for 2 years. Once dissolved, the solution can be stored at −20°C for 1 month and remains stable for 7 days at room temperature. In our experiment, we prepared a .5 g/L L‐NAME solution in real time by dissolving the powder in SPF animal drinking water. The solution was changed every 5 days throughout the study. Finally, the effect of L‐NAME on blood pressure in mice was assessed.^24^

### 2.4 Chemicals and reagents

DMEM and FBS were sourced from Gibco, while collagenase, trypsin, palmitic acid and fatty acid‐free BCA were obtained from Sigma. High‐fat feed (60%) was purchased from the Guangdong Animal Experiment Centre. The antibodies used in this study included anti‐PINK1 (Santa Cruz, sc‐517353, rabbit) and the following from Proteintech: anti‐Prdx2 (10545‐2‐AP, rabbit), anti‐P‐JNK (80024‐1‐RR, rabbit), anti‐P‐p38 (28796‐1‐AP, rabbit), anti‐JNK (24164‐1‐AP, rabbit), anti‐p38 (14064‐1‐AP, rabbit), anti‐Bcl2 (26593‐1‐AP, rabbit), anti‐Bax (50599‐2‐Ig, rabbit), anti‐C‐Caspase3 (68773‐1‐Ig, mouse), anti‐GFP (50430‐2‐AP, rabbit), anti‐ANP (27426‐1‐AP, rabbit) and anti‐β‐actin (66009‐1‐Ig, mouse).

### 2.5 Detection of LDs via fluorescence microscopy

To visualize LDs, NRCMs were isolated and inoculated on confocal cell dishes for 3–5 days and were incubated with 2 g/mL BODIPY 493/503. Fluorescent labelling was stopped by washing with PBS, and then immediately fixed with 4% paraformaldehyde. The image scale was set to 20 µm, and the green fluorescence intensity represents the amount of LDs.

### 2.6 ROS determination

Dihydroethidium (DHE; Sigma) staining of tissue sections and 2′,7′‐dichlorodihydrofluorescein diacetate (DCFH‐DA; Beyotime) staining of cells were used to assess ROS production. According to previously established protocols,^15^ harvested hearts were embedded in OCT, frozen at −80°C and sectioned using a cryostat. The sections were thawed and incubated with DHE staining for 30 min, followed by three PBS washes. Intracellular ROS levels of cells were quantified using a DCFH‐DA assay. Cells were incubated with 10 µM DCFH‐DA for 30 min, after which they were stained with DAPI and followed by three PBS washes. All the above incubation processes were carried out at 37°C. Confocal images were acquired using a Leica SP8 microscope, and analysis was performed using ImageJ software. Red fluorescence (DHE; scale bar 100 µm) and green fluorescence (DCFH‐DA; scale bar 50 µm) indicate the levels of ROS.

### 2.7 TdT‐mediated dUTP nick‐end labelling (TUNEL) assay

A TUNEL assay (Beyotime) was conducted to assess cardiomyocyte apoptosis both in vivo and in vitro. Harvested hearts were embedded, sectioned, dewaxed and stained as instruction. At room temperature, both heart and cells were fixed with 4% formaldehyde for 15 min and permeabilized using .2% Triton X‐100 for 20 min. Afterward, the samples were incubated with TUNEL solution at 37°C for 30 min. DAPI staining was then applied, followed by three additional PBS washes. Images were obtained using a Leica SP8 confocal microscope, and analysis was performed with ImageJ software. The image scale was set to 100 µm, and red fluorescence indicates the level of apoptosis.

### 2.8 Mitochondrial membrane potential (MMP) analysis

JC‐1 mitochondrial membrane potential assay kit (Beyotime) was employed to access MMP. Cells were washed by PBS three times and stained with JC‐1 solution for 30 min. Then cells were stained with DAPI and followed by three PBS washes. Images were captured with confocal microscope. The green fluorescence represents reduced MMP and red fluorescence corresponds to active MMP. Image‐J was used for analysis and the scale bar represents 100 µm.

### 2.9 Statistical analysis

Data are presented as mean ± SEM. For comparisons between two groups, Student's *t*‐test was applied, while one‐way analysis of variance followed by Tukey's test was used for multiple group comparisons. Statistical significance was defined as *p* < .05, with all tests being two‐sided. Analyses were performed using SPSS version 25.

## 3 RESULTS

### 3.1 PINK1 deficiency exacerbates cardiac dysfunction and reduces Prdx2 expression in HFpEF mice

**FIGURE 1:**
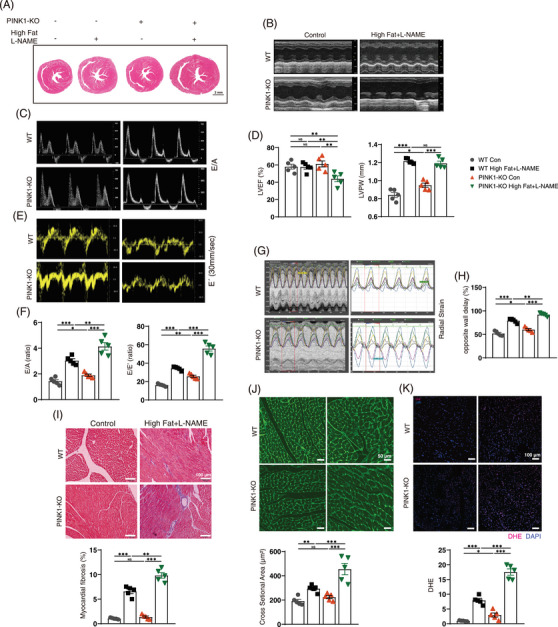
PTEN‐induced kinase 1 (PINK1) deficiency exacerbates myocardial reactive oxygen species (ROS) and aggravates cardiac dysfunction in heart failure with preserved ejection fraction (HFpEF) mice. (A) hematoxylin and eosin (H&E)‐stained heart sections. Scale bar, 1 mm. (B) Representative M‐mode echocardiographic images. (C) Representative E/A echocardiographic images. (D) Quantitative analyses of LVEF and LVPW. (E) Representative E´ echocardiographic images. (F) Quantitative analyses of E/A and E/E´. (G) Representative LV endocardial radical strain echocardiographic images. (H) Quantitative analyses of LV opposite wall delay. (I) Masson trichrome staining. Scale bar, 100 µm for Masson staining. (J) WGA staining. Scale bar, 50 µm. (K) Dihydroethidium (DHE) staining to reflect myocardial ROS production. Scale bar, 100 µm. For all statistical plots, the data are presented as the means ± SEs. *p < 0.05; **p < 0.01; ***p < 0.001 using Student *t* test.

To assess whether PINK deficiency exacerbates HFpEF, we induced HFpEF via a 60% high‐fat diet and L‐NAME for 15 weeks. Compared with control mice, HFpEF mice presented an increased body mass gain rate (Figure S1A), heart weight‐to‐body weight (HW/BW) ratio and lung weight‐to‐heart weight ratio (LW/HW) (Figure S1B). HFpEF mice had higher systolic blood pressure (SBP), serum glucose, serum cholesterol and triglyceride (TG) levels than control mice (Figure S1B,C). These factors were re‐evaluated in the PINK1‐KO‐HFpEF mice; the final body weights of the PINK1‐KO‐HFPEF mice were reduced, which resulted in an increase in HW/BW. Serum blood glucose and TG levels were greater in PINK1‐KO‐HFPEF group than WT‐HFpEF group (Figure S1B,C). Cardiac function was assessed via echocardiography after the 15 weeks induction of HFpEF. Compared with those in the control group, HFpEF mice exhibited diastolic dysfunction, represented by the increasing ratio between mitral E wave to A wave (E/A) and the mitral E wave to E´ wave (E/E´) (Figure 1C–F). In addition, the left ventricular posterior wall (LVPW) thickness and the opposite wall delay indicated severe left ventricular remodelling (Figure 1B,G). What is more, PINK1 deficiency further exacerbated diastolic dysfunction and left ventricular remodelling in HFpEF mice. Left ventricular ejection fraction was not significantly different between WT control and HFpEF mice but was distinctly reduced in PINK1‐deficient HFpEF mice (Figure 1B,D). HFpEF mice exhibited elevated serum BNP than control mice, and PINK1 deficiency further exacerbated BNP levels in HFpEF mice relative to WT mice (Figure S1C), as shown by ELISA. Moreover, serum IFN‐β, IL‐6, TGF‐β and TNFα levels were elevated in HFpEF group than WT control group, and PINK1 deficiency further increased these factors in HFpEF mice (Figure S1C). Masson's staining and wheat germ agglutinin stain (WGA) staining revealed that PINK1 deficiency exacerbated cardiac fibrosis and LV remodelling (Figure 1I,J). In addition, DHE staining revealed that ROS levels were greater in PINK1‐deficient HFpEF group (Figure 1K). These results further confirmed that PINK1 deficiency exacerbates HFpEF‐induced cardiac dysfunction in mice.

**FIGURE 2:**
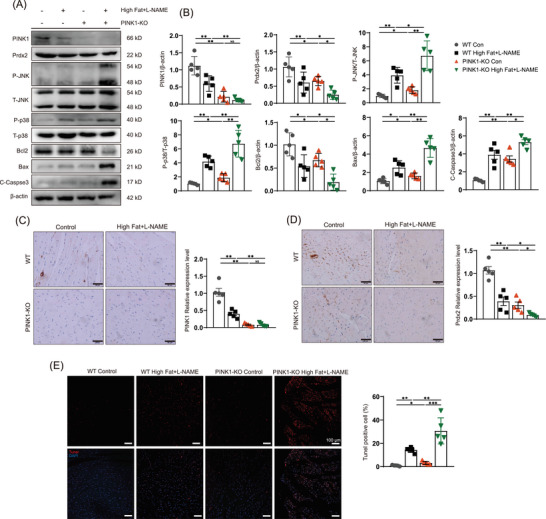
PTEN‐induced kinase 1 (PINK1) deficiency reduces peroxiredoxin‐2 (Prdx2) and exacerbates JNK and p38 phosphorylation leading to myocardial apoptosis. (A) Western blotting for the expression of PINK1, Prdx2, phospho‐p38, p38, phospho‐JNK, JNK, Bcl2, Bax, Cleaved‐Caspase3 and β‐actin in heart tissues. (B) Quantitative analyses of protein expression of PINK1, Prdx2, phospho‐p38, p38, phospho‐JNK, JNK, Bcl2, Bax, Cleaved‐Caspase3 and β‐actin. (C) immunohistochemical of PINK1 in heart tissues of WT and PINK1‐KO mice. Scale bar, 50 µm. (D) immunohistochemical of Prdx2 in heart tissues of WT and PINK1‐KO mice. Scale bar, 50 µm. (E) The apoptosis rate of cardiomyocytes by TUNEL assay Scale bar, 100 µm. For all statistical plots, the data are presented as the means ± SEs. *p < 0.05; **p < 0.01; ***p < 0.001 using Student *t* test.

Next, we examined protein expression in PINK1‐deficient HFpEF mice and found significantly decreased protein expression of Prdx2. Surprisingly, PINK1 deficiency affected Prdx2 expression (Figure 2A), as shown by immunoblotting. We subsequently examined PINK1 and Prdx2 expression and confirmed the results via immunohistochemistry (Figure 2C,D). To explore the impact of the reduction in Prdx2, we examined several downstream proteins associated with changes in Prdx2 by western blotting. Our findings revealed an increase in JNK and p38 phosphorylation during HFpEF and even more pronounced alterations in PINK1‐deficient HFpEF mice (Figure 2A). B‐cell lymphoma 2 (bcl2) was decreased, and increases in Bcl‐2‐associated X protein (Bax) and cleaved caspase‐3 cysteine protease (c‐caspase 3) were also observed (Figure 2A). We further performed TUNEL staining to compare apoptosis between four groups (Figure 2E), which confirmed an increase in apoptosis in PINK1‐deficient mice in the HFpEF group.

### 3.2 PINK1 overexpression increases Prdx2 and attenuates cardiac diastolic dysfunction in HFpEF mice

**FIGURE 3:**
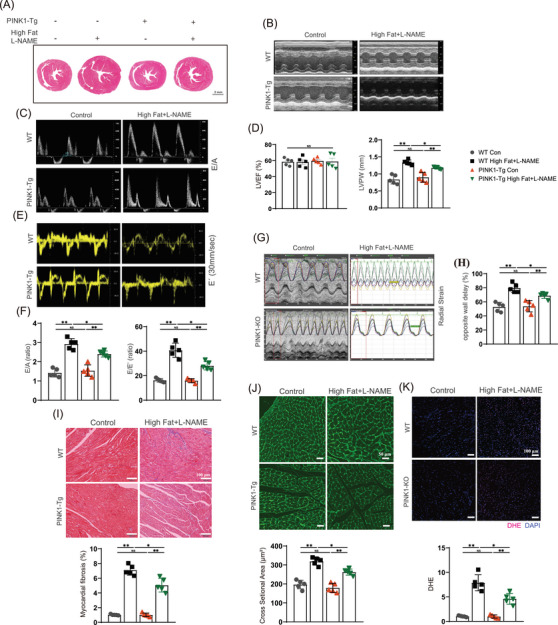
PTEN‐induced kinase 1 (PINK1) overexpression reduces myocardial reactive oxygen species (ROS) and attenuates heart failure with preserved ejection fraction (HFpEF) cardiac dysfunction. (A) hematoxylin and eosin (H&E)‐stained heart sections. Scale bar, 1 mm. (B) Representative M‐mode echocardiographic images. (C) Representative E/A echocardiographic images. (D) Quantitative analyses of LVEF and LVPW. (E) Representative E´ echocardiographic images. (F) Quantitative analyses of E/A and E/E´. (G) Representative LV endocardial radical strain echocardiographic images. (H) Quantitative analyses of LV opposite wall delay. (I) Masson trichrome staining. Scale bar, 100 µm for Masson staining. (J) WGA staining. Scale bar, 50 µm. (K) Dihydroethidium (DHE) staining to reflect myocardial ROS production. Scale bar, 100 µm. For all statistical plots, the data are presented as the means ± SEs. *p < 0.05; **p < 0.01; ***p < 0.001 using Student *t* test.

**FIGURE 4:**
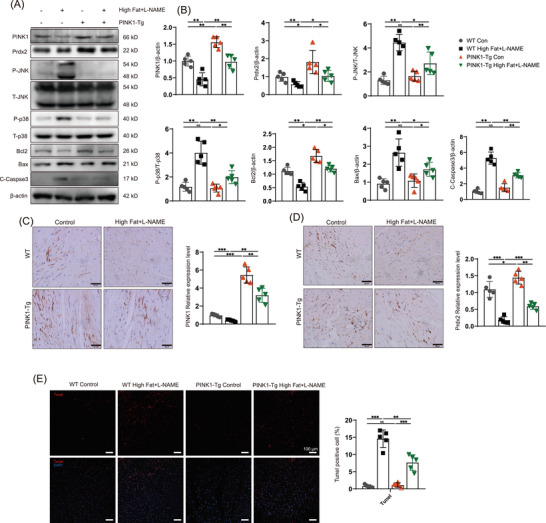
PTEN‐induced kinase 1 (PINK1) overexpression attenuates myocardial apoptosis by restoring peroxiredoxin‐2 (Prdx2). (A) Western blotting for the expression of PINK1, Prdx2, phospho‐p38, p38, phospho‐JNK, JNK, Bcl2, Bax, Cleaved‐Caspase3 and β‐actin in heart tissues. (B) Quantitative analyses of protein expression of PINK1, Prdx2, phospho‐p38, p38, phospho‐JNK, JNK, Bcl2, Bax, Cleaved‐Caspase3 and β‐actin. (C) immunohistochemical of PINK1 in heart tissues of WT and PINK1‐KO mice. Scale bar, 50 µm. (D) immunohistochemical of Prdx2 in heart tissues of WT and PINK1‐KO mice. Scale bar, 50 µm. (E) The apoptosis rate of cardiomyocytes by TUNEL assay Scale bar, 100 µm. For all statistical plots, the data are presented as the means ± SEs. *p < 0.05; **p < 0.01; ***p < 0.001 using Student *t* test.

We further investigated the effect of PINK1 overexpression following a high‐fat diet and L‐NAME induction. Therefore, we constructed transgenic mice that overexpressed PINK1 (PINK1‐Tg) and compared cardiac function and Prdx2 protein expression among the groups. PINK1‐Tg mice presented superior diastolic function and improved cardiac remodelling compared to WT mice after HFpEF induction by a high‐fat diet and L‐NAME. E/A, E/E´, opposite wall delay and LVPW in HFpEF mice were partially rescued by PINK overexpression (Figure 3B–H). Masson and WGA staining revealed that cardiac fibrosis and left ventricular remodelling were attenuated in the PINK1‐Tg mice (Figure 3I,J). In addition, less oxidative stress was observed in the HFpEF hearts of the PINK1‐Tg mice than in those of the WT mice (Figure 3K). The PINK1‐Tg‐HFpEF mice presented improved HW/BW, LW/HW and SBP (Figure S2A,B), and the serum levels of BNP, IFN‐β, IL‐6, TGF‐β and TNF‐α were notably lower in the PINK1‐Tg mice than in the WT mice (Figure S2C). Immunoblotting revealed an increase in Prdx2 in the PINK1‐Tg‐HFpEF mice (Figure 4A), which was accompanied by the attenuation of cardiac apoptosis. Immunohistochemistry and TUNEL staining further supported these findings (Figure 4C–E). These results suggest that PINK1 may alleviate cardiomyocyte apoptosis via Prdx2, thereby improving cardiac diastolic function.

### 3.3 PINK1 interacts with Prdx2, and both are impaired in palmitic acid‐induced lipotoxicity

**FIGURE 5:**
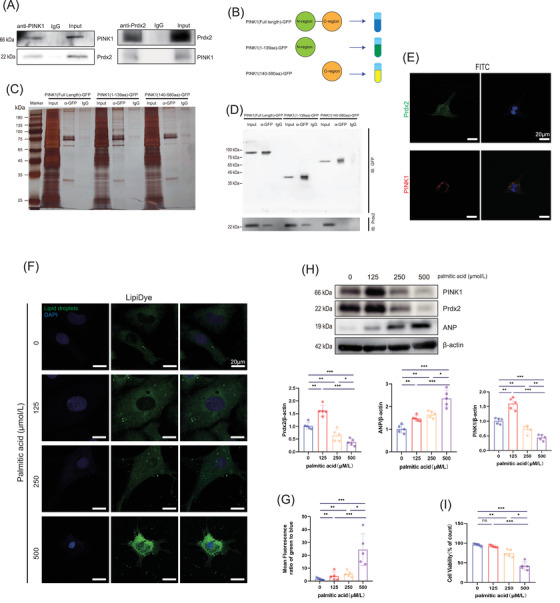
PTEN‐induced kinase 1 (PINK1) interacts with peroxiredoxin‐2 (Prdx2) and both are impaired in lipotoxicity. (A) Immunoprecipitation of PINK1 with Prdx2 in NRCM. (B) two alpha structure of PINK1: N‐terminal region (amino acids 1133) and C‐terminal region (amino acids 138500). (C) Detection of immunoprecipitation protein bands in a gel by silver staining. (D) Immunoblot of immunoprecipitation for the GFP and Prdx2. (E) Immunofluorescence co‐localisation of PINK1 with Prdx2 in NRCM. Scale bar, 25 µm. (F) Detection of LDs by Fluorescent Microscopy in palmitic acid gradient concentration. Scale bar, 25 µm. (G) Quantitative analyses of LDs. (H) Western blotting for the expression of PINK1, Prdx2, ANP and β‐actin in palmitic acid gradient concentration. (I) Cell viability of NRCM in palmitic acid gradient concentration. For all statistical plots, the data are presented as the means ± SEs. *p < 0.05; **p < 0.01; ***p < 0.001 using Student *t* test.

To identify the PINK1 interactome, we immunoprecipitated mouse heart tissue with an anti‐PINK1 antibody and performed mass spectrometry to analyse the obtained proteins. A collection of the proteins targeted by PINK1 is provided in Table S1. Intriguingly, the proteins targeted by PINK1 included Prdx2 peptides. To verify the results obtained by mass spectrometry, we performed endogenous co‐IP and found that PINK1 was associated with Prdx2 (Figure 5A). We next examined the colocalization of PINK1 with Prdx2 via immunofluorescence analysis (Figure 5E) and found that PINK1 colocalized with Prdx2 in NRCMs. These results suggest that PINK1 binds to Prdx2. According to the UniProt database,^25^ the supersecond structure of PINK1 includes two alpha helices: the N‐terminal region (amino acids 1–133) and the C‐terminal region (amino acids 138–500) (Figure S6A). Moreover, the two structures are predicted to be protein modification sites that interact with the other proteins. To identify the structural component of PINK1 that interacts with Prdx2, H9C2 cells were transfected with PINK1 (full‐length)‐GFP, PINK1 (1‐139 aa)‐GFP and PINK1 (140‐580 aa)‐GFP plasmids (Figure 5B). The cells were collected after 48 h for coimmunoprecipitation (co‐IP) experiments. After affinity purification, mass spectrometry was used to identify differences in the obtained proteins among the three groups (Table S2), and PEAKS software was used to search the protein database. Additionally, Figure S7 displays the abundance of the obtained proteins in the biological process, cellular component and molecular function categories. According to the mass spectrometry data, distinct signals of the Prdx2 protein were observed in the full‐length PINK1 and 1–139 aa PINK1 groups but were absent in the 140–580 aa PINK1 group (Figure S6B and Table S2). The areas enriched with the Prdx2 peptide were essentially identical between the PINK1 (full‐length) and PINK1 (1‐139 aa) groups (2.28 × 10^6^ and 2.44 × 10^6^) but significantly lower in the PINK1 (140–580 aa) group (1.62 × 10^6^) (Figure S6B and Table S2). These data suggest that the interaction between PINK1 and Prdx2 primarily occurs within the N‐terminal region. To verify the results obtained by mass spectrometry, we examined the endogenous interactions of the three groups. Proteins were coimmunoprecipitated with anti‐GFP (Figure 5C), and the pulldown samples were immunoblotted with GFP and Prdx2 antibodies. Immunoblotting revealed that Prdx2 was captured by the PINK1 (full length) and PINK1 (1–139 aa) groups (Figure 5D), which suggested that the N‐terminal region (amino acids 1–133) of PINK1 interacts directly with Prdx2.

To determine the appropriate concentration for myocardial lipotoxicity, we treated NRCMs with increasing concentrations (0, 125, 250 and 500 µmol/L) of palmitic acid for 24 h. Lipid droplet staining revealed a large increase in the number of intracellular droplets in response to 500 µmol/L palmitic acid (Figure 5F). We then examined PINK1 and Prdx2 protein expression and found that both PINK1 and Prdx2 showed similar trends: an initial increase in response to 125 µmol/L, followed by a decrease as palmitic acid content continued to increase (Figure 5H). In addition, ANP expression increased, and cell viability gradually decreased with increasing palmitic acid concentration (Figure 5H,I). Thus, 500 µmol/L palmitic acid was selected to induce myocardial lipotoxicity to examine the function of PINK1.

### 3.4 PINK1 ablation increases palmitic acid‐induced ROS production and apoptosis by reducing Prdx2

**FIGURE 6:**
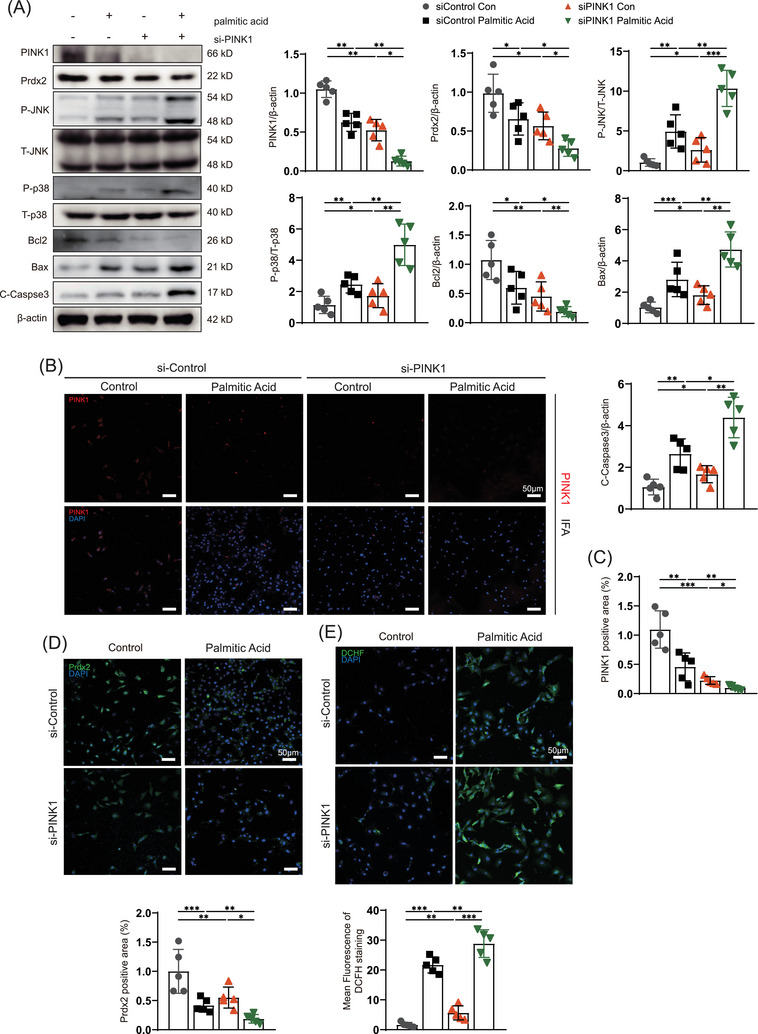
siPINK1 aggravates neonatal rat cardiomyocyte (NRCM) lipotoxicity through peroxiredoxin‐2 (Prdx2) reduction. (A) Western blotting for the expression of PINK1, Prdx2, phospho‐p38, p38, phospho‐JNK, JNK, Bcl2, Bax, Cleaved‐Caspase3 and β‐actin in myocardial lipotoxicity. (B) immunofluorescence of PINK1 in myocardial lipotoxicity. (C) Quantitative analyses of PINK1 positive area. (D) immunofluorescence of Prdx2 in myocardial lipotoxicity. (E) DCHF staining to reflect myocardial ROS production. Scale bar, 50 µm. For all statistical plots, the data are presented as the means ± SEs. *p < 0.05; **p < 0.01; ***p < 0.001 using Student *t* test.

To determine whether PINK1 ameliorates apoptosis in the context of myocardial lipotoxicity, we used siPINK1‐ and adPINK1‐infected NRCMs treated with 500 µmol/L palmitic acid. We examined the expression of apoptosis‐associated proteins and found that palmitic acid reduced the expression of PINK1 and Prdx2 and stimulated ROS production (Figure 6A,E). TUNEL staining revealed that apoptosis in NRCMs increased significantly after palmitic acid treatment (Figure S3B). After NRCMs were transfected with siPINK1, western blot and immunofluorescence analyses confirmed that siPINK1 inhibited PINK1 expression (Figure 6A,B). The expression of Prdx2 was also reduced as PINK1 expression decreased (Figure 6A). The increases in the P‐JNK/JNK and P‐p38/p38 ratios in response to myocardial lipotoxicity were significantly exacerbated by the reduction in Prdx2 (Figure 6A). Subsequent immunoblotting revealed the upregulation of Bax and caspase‐3 and the downregulation of Bcl‐2, which indicated activated apoptotic flux in palmitic acid‐treated NRCMs (Figure 6A) that was greatly exacerbated by siPINK1 transfection. DCHF‐DA staining revealed that siPINK1 exacerbated palmitic acid‐induced increases in ROS levels (Figure 6E). TUNEL and JC‐1 staining further indicated that siPINK1 increased lipotoxicity‐induced myocardial apoptosis and decreased the MMP (Figure S3A,B).

### 3.5 PINK1 overexpression attenuates palmitic acid‐induced ROS production and apoptosis by increasing Prdx2

**FIGURE 7:**
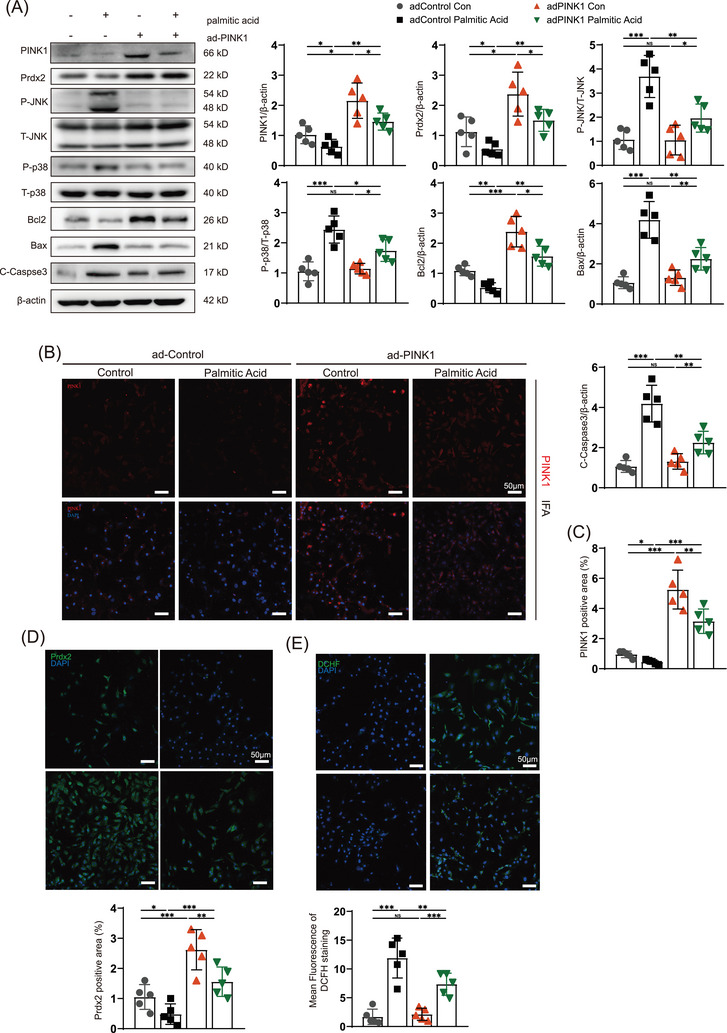
adPINK1 attenuates neonatal rat cardiomyocyte (NRCM) lipotoxicity by increasing peroxiredoxin‐2 (Prdx2). (A) Western blotting for the expression of PINK1, Prdx2, phospho‐p38, p38, phospho‐JNK, JNK, Bcl2, Bax, Cleaved‐Caspase3 and β‐actin in myocardial lipotoxicity. (B) immunofluorescence of PINK1 in myocardial lipotoxicity. (C) Quantitative analyses of PINK1 positive area. (D) immunofluorescence of Prdx2 in myocardial lipotoxicity. (E) DCHF staining to reflect myocardial ROS production. Scale bar, 50 µm. For all statistical plots, the data are presented as the means ± SEs. *p < 0.05; **p < 0.01; ***p < 0.001 using Student *t* test.

In contrast, the palmitic acid‐induced increase in ROS production and apoptosis in NRCMs was significantly reversed by adPINK1 transfection. Immunofluorescence and immunoblotting revealed that the palmitic acid‐induced disordered expression of Prdx2 was reversed by PINK1 overexpression (Figure 7A–D); P‐JNK/JNK and P‐p38/p38 were also suppressed (Figure 7A). Bcl2 was increased and Bax and C‐Caspase3 were decreased in palmitic acid‐treated NRCMs transfected with adPINK1 (Figure 7A), which indicated that apoptotic flux was obstructed. Moreover, DCFH‐DA indicated high levels of ROS generation in palmitic acid‐treated NRCMs (Figure 7E), which was improved by adPINK1 treatment. In addition, transfection with adPINK1 restored palmitic acid‐induced apoptotic flux and mitochondrial dysfunction, as shown by TUNEL and JC‐1 staining (Figure S4A,B), which was consistent with the immunoblot results. Furthermore, an analysis of Prdx2 gene expression and a cycloheximide chase (CHX) assay under PINK1 overexpression intervention revealed that while adPINK1 does not significantly affect Prdx2 mRNA levels, it notably enhances the stability of the Prdx2 protein (Figure S10A,B). The results from the CHX assay provide further evidence of PINK1's involvement in the regulation of Prdx2 at the protein level. These results revealed that PINK1 protected the myocardium from lipid toxicity via Prdx2, which reduced ROS production and apoptosis.

### 3.6 Prdx2 ablation attenuates PINK1 overexpression‐mediated alleviation of palmitic acid‐induced lipotoxicity

**FIGURE 8:**
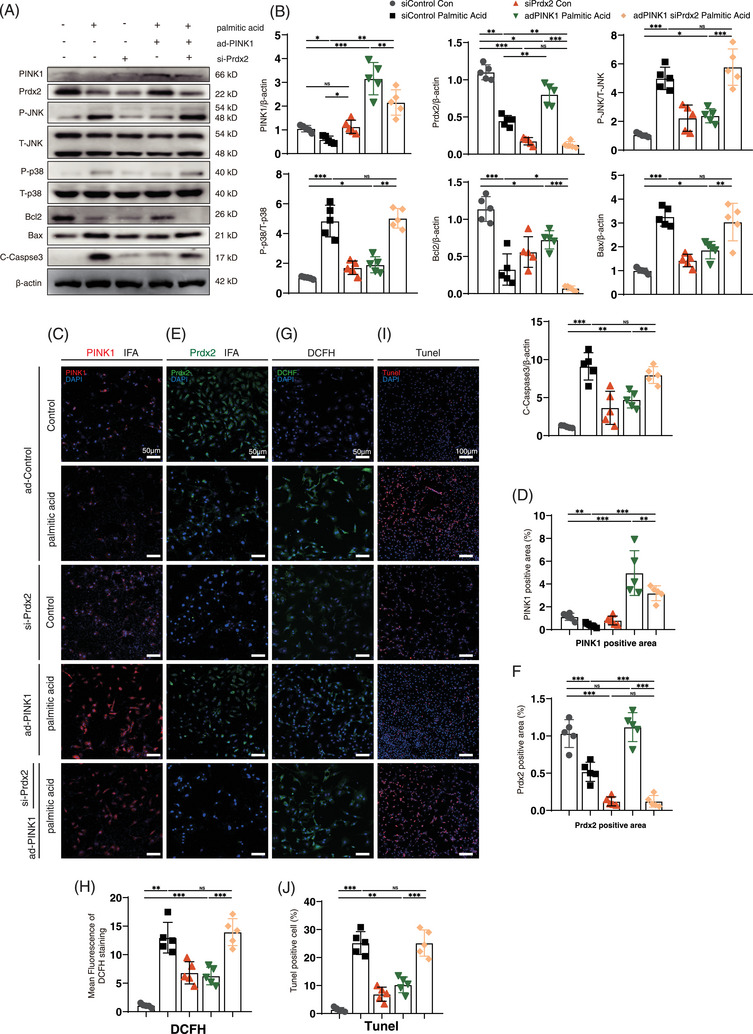
siPrdx2 attenuates the alleviating effect of adPINK1 on neonatal rat cardiomyocyte (NRCM) lipotoxicity. (A) Western blotting for the expression of PINK1, Prdx2, phospho‐p38, p38, phospho‐JNK, JNK, Bcl2, Bax, Cleaved‐Caspase3 and β‐actin in myocardial lipotoxicity. (B) Quantitative analyses of protein expression of PINK1, Prdx2, phospho‐p38, p38, phospho‐JNK, JNK, Bcl2, Bax, Cleaved‐Caspase3 and β‐actin. (C) immunofluorescence of PINK1 in myocardial lipotoxicity. (D) Quantitative analyses of PINK1 positive area. (E) immunofluorescence of Prdx2 in myocardial lipotoxicity. (F) Quantitative analyses of Prdx2 positive area. (G) DCHF staining to reflect myocardial ROS production. Scale bar, 50 µm. (H) Quantitative analyses of DCFH staining. (I) The apoptosis rate of cardiomyocytes by TUNEL assay. Scale bar, 100 µm. (J) Quantitative analyses of TUNEL positive area. For all statistical plots, the data are presented as the means ± SEs. *p < 0.05; **p < 0.01; ***p < 0.001 using Student *t* test.

To confirm the role of Prdx2 in the mechanism by which PINK1 counteracts lipotoxicity, NRCMs were transfected with adPINK1 and siPrdx2, and ROS production and apoptosis flux were examined after palmitic acid treatment. As expected, western blot analysis revealed that Prdx2 blockade reactivated the P‐JNK and P‐p38 pathways (Figure 8A). Pretreatment with siPrdx2 efficiently reversed the adPINK1‐mediated reduction in ROS levels (Figure 8G), as shown by DCFH staining. Moreover, TUNEL staining revealed a decrease in the antiapoptotic effect of adPINK1 after siPrdx2 pretreatment (Figure 8I), which suggested that Prdx2 is essential for PINK1‐mediated reductions in ROS production and apoptosis. JC‐1 staining also revealed a decrease in the MMP (Figure S5A), which suggested that adPINK1 induced impaired cellular energy metabolism after siPINK1 pretreatment. Taken together, these findings suggest that Prdx2 is important for the PINK1‐mediated alleviation of myocardial lipotoxicity.

To further confirm the role of mitochondrial ROS in Prdx2 ablation‐induced apoptosis, we performed additional experiments treating cells with palmitic acid and siPrdx2 to induce oxidative stress, while using SS‐31 to reduce mitochondrial ROS. MitoSOX staining and confocal fluorescence microscopy revealed that SS‐31 significantly attenuated the increase in mitochondrial ROS induced by siPrdx2 (Figure S12A). Moreover, SS‐31 reduced malondialdehyde levels elevated by siPrdx2 (Figure S12B). Additionally, we examined the levels of key apoptotic proteins, including Prdx2, Bcl‐2, Bax and cleaved Caspase‐3. Our results demonstrated that SS‐31 effectively decreased mitochondrial ROS levels and blocked the apoptotic effects induced by siPrdx2 (Figure S13), supporting the conclusion that mitochondrial ROS plays a significant role in Prdx2 reduction‐induced apoptosis.

## 4 DISCUSSION

HFpEF represents a significant public health challenge, yet the functional role of PINK1 in this condition remains unclear. Our findings suggest that the N‐terminal region (amino acids 1–133) of PINK1 interacts with Prdx2 to inhibit lipotoxicity‐induced myocardial apoptosis and improve cardiac diastolic function. In this study, we established an HFpEF model, and consistent with the literature,^26^ severe cardiac diastolic dysfunction was observed. Additionally, impaired PINK1 and Prdx2 expression was observed in HFpEF mice, leading to the activation of apoptotic signalling pathways (JNK and p38). This activation, exacerbated by decreased Prdx2, contributed to increased fibrosis, inflammation and cardiac dysfunction. We further demonstrated that PINK1 deficiency inhibited Prdx2 expression and drove the overactivation of JNK and p38 signalling and accelerating cardiac dysfunction. Conversely, PINK1 overexpression restored Prdx2 expression and decreased ROS production and apoptosis, which reduced fibrosis and inflammation, ultimately improved cardiac function in HFpEF mice.

We further identified an interaction between PINK1 and Prdx2 in vitro, which suggested that PINK1 contributed to a reduction in apoptosis though Prdx2. Synchronized changes in myocardial lipotoxicity associated with PINK1 and Prdx2 were also observed. Our findings revealed that the N‐terminal region (amino acids 1–133) of PINK1 interacts with Prdx2 and subsequently reduces the phosphorylation of JNK and MAPK, which decreased apoptotic proteins, such as Bax and Caspase3, and ultimately mitigating myocardial lipotoxicity‐induced apoptosis. Furthermore, Prdx2 ablation attenuated the suppression of the JNK and MAPK pathways by PINK1 and subsequently reactivated apoptotic flux, which compromised the ability of PINK1 to alleviate myocardial injury.

HFpEF is characterized by multiple systemic abnormalities and intricate pathophysiological mechanisms, making it a complex condition with unique challenges. Lipotoxicity is a crucial contributor to hypertrophic remodelling and cardiac dysfunction.^27^, ^28^ Clinical observations validated the association between lipotoxicity and diastolic dysfunction. A CMR assessment of intramyocardial fat content among HFpEF, HFrEF and control subjects revealed greater than twofold lipid accumulation in HFpEF hearts than in control hearts.^5^, ^29^ Myocardial lipotoxicity is a primary pathophysiological mechanism underlying HFpEF,^8^, ^10^, ^30^, ^31^, ^32^ while PINK1's role remains poorly understood.^4^, ^33^ In our study, PINK1 was elevated in response to low concentrations of palmitic acid and decreased in response to high concentrations of palmitic acid, indicating impaired PINK1 expression during lipotoxicity. In vitro, siPINK1 administration to NRCMs increased ROS production and myocardial apoptosis in response to lipotoxicity. PINK1 overexpression attenuated lipotoxicity‐induced myocardial apoptosis and improved mitochondrial metabolism. These findings provide evidence that PINK1 attenuates myocardial injury induced by lipotoxicity.

A previous study demonstrated that PINK1 was transiently activated and then downregulated in cardiac tissues via the progression of HF and that the restoration of PINK1 attenuated cardiac dysfunction induced by pressure overload.^16^ Moreover, PINK1 deficiency in mice exacerbated angiotensin II‐induced cardiac hypertrophy and compromised cardiomyocyte mitochondrial function.^17^ Consistently, in our earlier research, we demonstrated that PINK1 deficiency exacerbated angiotensin II‐induced cardiac hypertrophy and impaired cardiomyocyte mitochondrial function, which was associated with HFrEF exacerbation.^15^ Our study revealed that PINK1 expression was decreased after 15 weeks of a high‐fat diet and L‐NAME, indicating a potential role for PINK1 in the progression of HFpEF. In vivo, PINK1 deficiency increased ROS production, promoted apoptosis and fibrosis and ultimately exacerbated HFpEF‐induced cardiac dysfunction. In contrast, PINK1 overexpression protected against ROS production and apoptosis, thereby improving cardiac diastolic function during HFpEF. Taken together, our results suggest that PINK1 protects the heart against diastolic dysfunction in HFpEF mice.

In addition to the effect of PINK1 on Parkin ubiquitination, studies have suggested that PINK1 affects or is affected by multiple proteins to result in various biological effects. For example, AMPKα2 protects against the development of HF via PINK1 phosphorylation.^17^ Moreover, PINK1 regulates p53 activation to suppress colon tumour growth.^34^ Most recently, emerging evidence has shown that PINK1 is related to the Prdx family and protects against cardiovascular disease.^18^ The Prdx family has emerged as a key player in the development of cardiovascular disease, particularly Prdx2, which effectively scavenges cellular ROS and reduces apoptosis. Our study revealed the relationship between PINK1 and Prdx2 and revealed that PINK1 reduced cardiomyocyte apoptosis, counteracted lipotoxicity and improved cardiac function via Prdx2. These findings provide new evidence for the biological effect of PINK1.

Mammalian Prdx proteins are ubiquitously distributed within cells and perform a variety of biological functions.^19^ The Prdx family has been implicated in cardiovascular disease, and Prdx1 prevents pressure overload‐induced HF,^35^ whereas Prdx3 overexpression inhibits left ventricular remodelling and failure after myocardial infarction.^18^ Prdx2 is a member of the Prdx family, which possesses peroxidase activity and plays a vital role in the removal of ROS, including hydrogen peroxide (H_2_O_2_), organic hydroperoxides and peroxynitrite.^36^ Prdx2‐deficient mice suffer from cardiac injury.^37^, ^38^ The downregulation of Prdx2 has been implicated in isoproterenol‐induced cardiac hypertrophy.^39^ A recent study showed that Prdx2 overexpression attenuated cardiac microvascular injury in diabetes by inhibiting mitochondria‐associated ferroptosis.^40^ Another study confirmed that Prdx2 deficiency increases oxidative stress and inflammatory responses and accelerates angiotensin II‐induced abdominal aortic aneurysm.^38^ Our study revealed that Prdx2 upregulation inhibited lipotoxicity‐induced ROS production and apoptosis, thus reducing cardiac injury in HFpEF mice.

Decreased Prdx2 expression represents a major risk factor for instigating apoptosis.^31^, ^41^ The mechanisms of Prdx2‐induced apoptosis include the activation of the JNK and p38 pathways and the liberation of cytochrome c triggered by ROS.^42^ These mechanisms initiate both caspase‐dependent and caspase‐independent apoptotic cascades to ultimately contribute to the onset and progression of HF.^42^ Earlier investigations suggested that the exposure of human cardiac progenitor cells to H_2_O_2_ induced apoptosis via the activation of the JNK signalling pathway.^31^ Prdx2 deficiency is associated with increased activation of JNK and p38 during accelerated plaque formation.^37^ For the first time, our study demonstrated the interaction between Prdx2 and PINK, which effectively restored Prdx2 expression and inhibited cardiomyocyte apoptosis by reducing the phosphorylation of JNK and MAPK, which improved cardiac diastolic function in HFpEF mice. These results provide new insights into the mechanistic effects of PINK1 and suggest a novel therapeutic target for treating HFpEF.

This study was subject to several limitations. First, we did not establish specific PINK1‐Prdx2 double‐knockout mice in vivo. Data from double‐knockout mice would be more convincing. Second, because HFpEF is a multifactorial disease that may activate multiple apoptotic pathways, the possibility that PINK1 protects cardiomyocytes from apoptosis via other pathways cannot be excluded. Collectively, our results indicate that PINK1‐induced Prdx2 expression might be a therapeutic target for HFpEF.

## 5 CONCLUSION

Lipotoxicity plays an important role in the pathogenesis of HFpEF. Our investigation reveals the dual functionality of PINK1, extending beyond its recognized role in mitochondrial autophagy. PINK1 has the capacity to increase Prdx2 expression, which leads to the efficient clearance of ROS. By modulating the JNK and p38 pathways, PINK1 effectively mitigates cardiomyocyte apoptosis, thereby alleviating myocardial lipotoxicity and ameliorating cardiac function in HFpEF. This comprehensive examination of PINK1 and Prdx2 provides valuable insights for innovative therapeutic approaches in the prevention and treatment of HFpEF

## AUTHOR CONTRIBUTIONS

Hao Zhang and Tianyu Xu designed the study. Xiyuan Mei, Qiling Yang and Qingchun Zeng carried out experiments. Xianghui Zeng, Zhuang Ma and Haobin Zhou analyzed and interpreted the data; Qingchun Zeng, Dingli Xu and Hao Ren drafted and revised the paper; all authors approved the final version of the paper.

## CONFLICT OF INTEREST STATEMENT

The authors declare no conflicts of interest.

## ETHICS STATEMENT

All animals were housed under pathogen‐free conditions. All protocols were approved by the Southern Medical University Institutional Animal Care and Use Committee (Approval no. K2019022). All procedures conformed to the NIH Guide for the Care and Use of Laboratory Animals.

## Supporting information



Supporting information

Supporting information

Supporting information

Supporting information

## Data Availability

All relevant data pertaining to this study are either provided in the article or available as Supporting Information.
