# Supplementary material for: PINK1 modulates Prdx2 to reduce lipotoxicity‐induced apoptosis and attenuate cardiac dysfunction in heart failure mice with a preserved ejection fraction
Source: Clin Transl Med. 2025 Jan 6;15(1):e70166. doi: 10.1002/ctm2.70166 (PMC11705485; doi:10.1002/ctm2.70166)
Supplement: Supplementary file 3 — Supporting information [file CTM2-15-e70166-s003.docx]

**serine/threonine-protein kinase PINK1, mitochondrial**

NCBI Reference Sequence: XP_032751497.1

LOCUS：XP_032751497 580 aa linear ROD 14-MAR-2020

<https://www.ncbi.nlm.nih.gov/protein/XP_032751497.1?report=fasta>

[PREDICTED: Rattus rattus PTEN induced kinase 1 (Pink1), mRNA - Nucleotide - NCBI (nih.gov)](https://www.ncbi.nlm.nih.gov/nuccore/XM_032895606.1)

**PINK1 full-length protein information**

GenPept Identical Proteins Graphics

>XP_032751497.1 serine/threonine-protein kinase PINK1, mitochondrial [Rattus rattus]

MAVRQALGRGLQLGRALLLRFAPKPGPVSGWGKPGPGAAWGRGERPGPVSSPGAQPRPLGLPLPDRYRFFRQSVAGLAARIQRQFVVRARGGAGPCGRAVFLAFGLGLGLIEEKQAESRRAASACQEIQAIFTQKNKQVSDPLDTRRWQGFRLEDYLIGQAIGKGCNAAVYEATMPTLPQHLEKAKHLGLLGKGPDVVSKGTDGEQAPGAPAFPFAIKMMWNISAGSSSEAILSKMSQELVPASRMALDGEYGAVTYRRSRDGPKQLAPHPNIIRVFRAFTSSVPLLPGALADYPDMLPPHYYPEGLGHGRTLFLVMKNYPCTLRQYLEEQTPSSRLATMMTLQLLEGVDHLVQQGIAHRDLKSDNILVEWDSDGCPWLVISDFGCCLADERVGLQLPFNSSSVERGGNGSLMAPEVSTAHSGPHAVIDYSKADTWAVGAIAYEIFGLANPFYGQGSAHLESRSYQEAQLPEMPKSVPPETRQLVRSLLQREASKRPSARIAANVLHLSLWGEHLLALKNLKLDKMIAWLLQQSAATLLADRLREKSCVETKLQMLFLANLECEALCQAALLLSSWRAAP

**1, Construction of Pink1 full-length protein overexpression plasmid-DNA sequence information;**；

Atggcggtgcgacaggcactgggccgaggcctgcagctgggtcgggcgctgctgctgcgcttcgctcccaagcccggcccggtgtcaggctggggc

Aagcccggccccggtgcggcctggggccgcggagagcgccccggcccggtctcaagcccgggagcacagccgcgtccgctcgggctccccctcccg

Gaccgctaccgcttcttccgccagtcggtggctgggctggcggcgcgaatccagcggcagttcgtggtgcgggcccgaggcggcgcagggccttgc

Ggccgagcagtcttcctggccttcgggctgggattggggttgatcgaggagaagcaggcggagagccggagggccgcctcggcctgtcaggagatc

Caggcaatttttacacagaaaaacaagcaagtgtctgacccactggacacacgacgttggcagggcttccgcctggaggattatctgataggacag

Gccatcggcaagggctgcaatgccgctgtgtatgaagccaccatgcccacgctgccccagcacttggagaaggccaaacaccttggccttctagga

Aaaggcccagatgtcgtctcaaagggaacagatggggagcaggctccaggggcccccgccttcccctttgccatcaaaatgatgtggaatatctcg

Gcaggttcctccagcgaagccatcttaagcaaaatgagccaggagctggtcccagcaagccgcatggctttggatggagagtatggagccgttact

Tacagaagatccagagacggtcccaagcagcttgccccgcaccccaacatcatccgggttttccgcgccttcacttcatctgtgcccctcctgccc

Ggggccctggctgactatcctgacatgctacccccacactactacccagaaggcctgggccacggccgcacactcttcctcgttatgaagaactac

Ccctgtaccctgcgccagtaccttgaagagcaaactcccagctcccgcctggctaccatgatgaccttgcagttactggagggcgtggaccacctc

Gttcagcagggcattgcccatcgagacctcaagtccgacaacatcctcgtggaatgggactcagatggctgcccctggctggtgatctccgacttt

Ggctgctgcctggctgatgagcgtgttggcctgcagttacctttcaacagctccagtgtagagcgtggtggcaatggctccctcatggcccctgag

Gtgtccacagcccattctggcccccatgcggtaattgactacagcaaggctgacacgtgggctgtgggggccatcgcctatgaaatcttcgggctt

Gccaaccccttctatggccaaggcagtgcccaccttgagagccgcagctaccaggaagctcagctgcccgagatgcccaagtcggtgcctccagaa

Acaagacagctggtgaggtccctgctccagcgcgaggccagcaagaggccgtctgcacgcatagctgccaacgtgctgcacttaagcctctggggt

Gagcatcttctagccctgaagaatctgaaattggacaagatgattgcctggctcctccagcagtcagcagccactctgctggctgacaggctgaga

Gagaagagctgcgtggagacgaagctgcagatgctgtttctggctaacctggagtgtgaggcactctgccaggcagccctcctcctctcttcctgg

agggcagccccatga

DNA- amino acids：

Atggcggtgcgacaggcactgggccgaggcctgcagctgggtcgggcgctgctgctgcgcttcgctcccaagcccggcccggtgtcaggctggggc

MAVRQALGRGLQLGRALLLRFAPKPGPVSGWG

Aagcccggccccggtgcggcctggggccgcggagagcgccccggcccggtctcaagcccgggagcacagccgcgtccgctcgggctccccctcccg

KPGPGAAWGRGERPGPVSSPGAQPRPLGLPLP

Gaccgctaccgcttcttccgccagtcggtggctgggctggcggcgcgaatccagcggcagttcgtggtgcgggcccgaggcggcgcagggccttgc

DRYRFFRQSVAGLAARIQRQFVVRARGGAGPC

Ggccgagcagtcttcctggccttcgggctgggattggggttgatcgaggagaagcaggcggagagccggagggccgcctcggcctgtcaggagatc

GRAVFLAFGLGLGLIEEKQAESRRAASACQEI

Caggcaatttttacacagaaaaacaagcaagtgtctgacccactggacacacgacgttggcagggcttccgcctggaggattatctgataggacag

QAIFTQKNKQVSDPLDTRRWQGFRLEDYLIGQ

Gccatcggcaagggctgcaatgccgctgtgtatgaagccaccatgcccacgctgccccagcacttggagaaggccaaacaccttggccttctagga

AIGKGCNAAVYEATMPTLPQHLEKAKHLGLLG

Aaaggcccagatgtcgtctcaaagggaacagatggggagcaggctccaggggcccccgccttcccctttgccatcaaaatgatgtggaatatctcg

KGPDVVSKGTDGEQAPGAPAFPFAIKMMWNIS

Gcaggttcctccagcgaagccatcttaagcaaaatgagccaggagctggtcccagcaagccgcatggctttggatggagagtatggagccgttact

AGSSSEAILSKMSQELVPASRMALDGEYGAVT

Tacagaagatccagagacggtcccaagcagcttgccccgcaccccaacatcatccgggttttccgcgccttcacttcatctgtgcccctcctgccc

YRRSRDGPKQLAPHPNIIRVFRAFTSSVPLLP

Ggggccctggctgactatcctgacatgctacccccacactactacccagaaggcctgggccacggccgcacactcttcctcgttatgaagaactac

GALADYPDMLPPHYYPEGLGHGRTLFLVMKNY

Ccctgtaccctgcgccagtaccttgaagagcaaactcccagctcccgcctggctaccatgatgaccttgcagttactggagggcgtggaccacctc

PCTLRQYLEEQTPSSRLATMMTLQLLEGVDHL

Gttcagcagggcattgcccatcgagacctcaagtccgacaacatcctcgtggaatgggactcagatggctgcccctggctggtgatctccgacttt

VQQGIAHRDLKSDNILVEWDSDGCPWLVISDF

Ggctgctgcctggctgatgagcgtgttggcctgcagttacctttcaacagctccagtgtagagcgtggtggcaatggctccctcatggcccctgag

GCCLADERVGLQLPFNSSSVERGGNGSLMAPE

Gtgtccacagcccattctggcccccatgcggtaattgactacagcaaggctgacacgtgggctgtgggggccatcgcctatgaaatcttcgggctt

VSTAHSGPHAVIDYSKADTWAVGAIAYEIFGL

Gccaaccccttctatggccaaggcagtgcccaccttgagagccgcagctaccaggaagctcagctgcccgagatgcccaagtcggtgcctccagaa

ANPFYGQGSAHLESRSYQEAQLPEMPKSVPPE

Acaagacagctggtgaggtccctgctccagcgcgaggccagcaagaggccgtctgcacgcatagctgccaacgtgctgcacttaagcctctggggt

TRQLVRSLLQREASKRPSARIAANVLHLSLWG

Gagcatcttctagccctgaagaatctgaaattggacaagatgattgcctggctcctccagcagtcagcagccactctgctggctgacaggctgaga

EHLLALKNLKLDKMIAWLLQQSAATLLADRLR

Gagaagagctgcgtggagacgaagctgcagatgctgtttctggctaacctggagtgtgaggcactctgccaggcagccctcctcctctcttcctgg

EKSCVETKLQMLFLANLECEALCQAALLLSSW

agggcagccccatga

RAAP

**2、1-139aa Protein Information**

MAVRQALGRGLQLGRALLLRFAPKPGPVSGWGKPGPGAAWGRGERPGPVSSPGAQPRPLGLPLPDRYRFFRQSVAGLAARIQRQFVVRARGGAGPCGRAVFLAFGLGLGLIEEKQAESRRAASACQEIQAIFTQKNKQV

**1-139aa DNA Information**

Atggcggtgcgacaggcactgggccgaggcctgcagctgggtcgggcgctgctgctgcgcttcgctcccaagcccggcccggtgtcaggctggggc

Aagcccggccccggtgcggcctggggccgcggagagcgccccggcccggtctcaagcccgggagcacagccgcgtccgctcgggctccccctcccg

Gaccgctaccgcttcttccgccagtcggtggctgggctggcggcgcgaatccagcggcagttcgtggtgcgggcccgaggcggcgcagggccttgc

Ggccgagcagtcttcctggccttcgggctgggattggggttgatcgaggagaagcaggcggagagccggagggccgcctcggcctgtcaggagatc

Caggcaatttttacacagaaaaacaagcaagtg

**1-139aa DNA-amino acids**：

Atggcggtgcgacaggcactgggccgaggcctgcagctgggtcgggcgctgctgctgcgcttcgctcccaagcccggcccggtgtcaggctggggc

MAVRQALGRGLQLGRALLLRFAPKPGPVSGWG

Aagcccggccccggtgcggcctggggccgcggagagcgccccggcccggtctcaagcccgggagcacagccgcgtccgctcgggctccccctcccg

KPGPGAAWGRGERPGPVSSPGAQPRPLGLPLP

Gaccgctaccgcttcttccgccagtcggtggctgggctggcggcgcgaatccagcggcagttcgtggtgcgggcccgaggcggcgcagggccttgc

DRYRFFRQSVAGLAARIQRQFVVRARGGAGPC

Ggccgagcagtcttcctggccttcgggctgggattggggttgatcgaggagaagcaggcggagagccggagggccgcctcggcctgtcaggagatc

GRAVFLAFGLGLGLIEEKQAESRRAASACQEI

Caggcaatttttacacagaaaaacaagcaagtg

QAIFTQKNKQV

**3、140-580 aa** **Protein Information**

SDPLDTRRWQGFRLEDYLIGQAIGKGCNAAVYEATMPTLPQHLEKAKHLGLLGKGPDVVSKGTDGEQAPGAPAFPFAIKMMWNISAGSSSEAILSKMSQELVPASRMALDGEYGAVTYRRSRDGPKQLAPHPNIIRVFRAFTSSVPLLPGALADYPDMLPPHYYPEGLGHGRTLFLVMKNYPCTLRQYLEEQTPSSRLATMMTLQLLEGVDHLVQQGIAHRDLKSDNILVEWDSDGCPWLVISDFGCCLADERVGLQLPFNSSSVERGGNGSLMAPEVSTAHSGPHAVIDYSKADTWAVGAIAYEIFGLANPFYGQGSAHLESRSYQEAQLPEMPKSVPPETRQLVRSLLQREASKRPSARIAANVLHLSLWGEHLLALKNLKLDKMIAWLLQQSAATLLADRLREKSCVETKLQMLFLANLECEALCQAALLLSSWRAAP

**140-580 aa Protein Information**：

tctgacccactggacacacgacgttggcagggcttccgcctggaggattatctgataggacag

Gccatcggcaagggctgcaatgccgctgtgtatgaagccaccatgcccacgctgccccagcacttggagaaggccaaacaccttggccttctagga

Aaaggcccagatgtcgtctcaaagggaacagatggggagcaggctccaggggcccccgccttcccctttgccatcaaaatgatgtggaatatctcg

Gcaggttcctccagcgaagccatcttaagcaaaatgagccaggagctggtcccagcaagccgcatggctttggatggagagtatggagccgttact

Tacagaagatccagagacggtcccaagcagcttgccccgcaccccaacatcatccgggttttccgcgccttcacttcatctgtgcccctcctgccc

Ggggccctggctgactatcctgacatgctacccccacactactacccagaaggcctgggccacggccgcacactcttcctcgttatgaagaactac

Ccctgtaccctgcgccagtaccttgaagagcaaactcccagctcccgcctggctaccatgatgaccttgcagttactggagggcgtggaccacctc

Gttcagcagggcattgcccatcgagacctcaagtccgacaacatcctcgtggaatgggactcagatggctgcccctggctggtgatctccgacttt

Ggctgctgcctggctgatgagcgtgttggcctgcagttacctttcaacagctccagtgtagagcgtggtggcaatggctccctcatggcccctgag

Gtgtccacagcccattctggcccccatgcggtaattgactacagcaaggctgacacgtgggctgtgggggccatcgcctatgaaatcttcgggctt

Gccaaccccttctatggccaaggcagtgcccaccttgagagccgcagctaccaggaagctcagctgcccgagatgcccaagtcggtgcctccagaa

Acaagacagctggtgaggtccctgctccagcgcgaggccagcaagaggccgtctgcacgcatagctgccaacgtgctgcacttaagcctctggggt

Gagcatcttctagccctgaagaatctgaaattggacaagatgattgcctggctcctccagcagtcagcagccactctgctggctgacaggctgaga

Gagaagagctgcgtggagacgaagctgcagatgctgtttctggctaacctggagtgtgaggcactctgccaggcagccctcctcctctcttcctgg

agggcagccccatga

**140-580 aa** **DNA-amino acids**：

tctgacccactggacacacgacgttggcagggcttccgcctggaggattatctgataggacag

SDPLDTRRWQGFRLEDYLIGQ

Gccatcggcaagggctgcaatgccgctgtgtatgaagccaccatgcccacgctgccccagcacttggagaaggccaaacaccttggccttctagga

AIGKGCNAAVYEATMPTLPQHLEKAKHLGLLG

Aaaggcccagatgtcgtctcaaagggaacagatggggagcaggctccaggggcccccgccttcccctttgccatcaaaatgatgtggaatatctcg

KGPDVVSKGTDGEQAPGAPAFPFAIKMMWNIS

Gcaggttcctccagcgaagccatcttaagcaaaatgagccaggagctggtcccagcaagccgcatggctttggatggagagtatggagccgttact

AGSSSEAILSKMSQELVPASRMALDGEYGAVT

Tacagaagatccagagacggtcccaagcagcttgccccgcaccccaacatcatccgggttttccgcgccttcacttcatctgtgcccctcctgccc

YRRSRDGPKQLAPHPNIIRVFRAFTSSVPLLP

Ggggccctggctgactatcctgacatgctacccccacactactacccagaaggcctgggccacggccgcacactcttcctcgttatgaagaactac

GALADYPDMLPPHYYPEGLGHGRTLFLVMKNY

Ccctgtaccctgcgccagtaccttgaagagcaaactcccagctcccgcctggctaccatgatgaccttgcagttactggagggcgtggaccacctc

PCTLRQYLEEQTPSSRLATMMTLQLLEGVDHL

Gttcagcagggcattgcccatcgagacctcaagtccgacaacatcctcgtggaatgggactcagatggctgcccctggctggtgatctccgacttt

VQQGIAHRDLKSDNILVEWDSDGCPWLVISDF

Ggctgctgcctggctgatgagcgtgttggcctgcagttacctttcaacagctccagtgtagagcgtggtggcaatggctccctcatggcccctgag

GCCLADERVGLQLPFNSSSVERGGNGSLMAPE

Gtgtccacagcccattctggcccccatgcggtaattgactacagcaaggctgacacgtgggctgtgggggccatcgcctatgaaatcttcgggctt

VSTAHSGPHAVIDYSKADTWAVGAIAYEIFGL

Gccaaccccttctatggccaaggcagtgcccaccttgagagccgcagctaccaggaagctcagctgcccgagatgcccaagtcggtgcctccagaa

ANPFYGQGSAHLESRSYQEAQLPEMPKSVPPE

Acaagacagctggtgaggtccctgctccagcgcgaggccagcaagaggccgtctgcacgcatagctgccaacgtgctgcacttaagcctctggggt

TRQLVRSLLQREASKRPSARIAANVLHLSLWG

Gagcatcttctagccctgaagaatctgaaattggacaagatgattgcctggctcctccagcagtcagcagccactctgctggctgacaggctgaga

EHLLALKNLKLDKMIAWLLQQSAATLLADRLR

Gagaagagctgcgtggagacgaagctgcagatgctgtttctggctaacctggagtgtgaggcactctgccaggcagccctcctcctctcttcctgg

EKSCVETKLQMLFLANLECEALCQAALLLSSW

agggcagccccatga

RAAP
