# Supplementary material for: PINK1 modulates Prdx2 to reduce lipotoxicity‐induced apoptosis and attenuate cardiac dysfunction in heart failure mice with a preserved ejection fraction
Source: Clin Transl Med. 2025 Jan 6;15(1):e70166. doi: 10.1002/ctm2.70166 (PMC11705485; doi:10.1002/ctm2.70166)
Supplement: Supplementary file 4 — Supporting information [file CTM2-15-e70166-s002.pdf]

Material and Method

1. Echocardiographic analysis

Echocardiographic measurements were performed to evaluate cardiac structure and function in the mice. briefly, echocardiography was conducted by Vevo 2100 system (Visual Sonics, Ontario, Canada), under body temperature-controlled conditions, 1.5% to 2% isoflurane was used to continuous anaesthesia during echocardiogram acquisition, isoflurane was adjusted to maintain a heart rate in the range of 400–500 beats per minute. Mice are gently restrained on the operation desk. The left ventricular ejection fraction (LVEF) and additional indices of systolic function were acquired from mid-ventricular level short-axis M-mode scans. Diastolic function measurements, utilizing pulsed-wave and tissue Doppler at the mitral valve level, were obtained from apical 4-chamber views. The parameters collected included LVEF, left ventricular end-diastolic posterior wall thickness (LVPW,d), peak Doppler blood inflow velocity during early diastole (E), peak Doppler blood inflow velocity during late diastole (A), and peak tissue Doppler velocity of myocardial relaxation at the mitral valve annulus during early diastole (E´). The maximum opposing wall delay was calculated by Vevo Strain software.

2. Histology and immunohistochemical staining

Fifteen weeks after HFD and L-NAME, the body weight of mice was recorded every four weeks. mice were sacrificed after anaesthetized with ketamine/xylazine (100/5 mg/kg). the heart and lung were collected for further analysis. The hearts were fixed in 10% formalin and embedded in paraffin according to standard histological

protocols. Embedded hearts were sectioned into 5-μm segments and stained with haematoxylin and eosin (H&E), Masson’ s trichrome, and wheat germ agglutinin (WGA). H&E and WGA staining were used to evaluate myocardial hypertrophy, and Masson’s trichrome staining was used to assess myocardial fibrosis. For immunohistochemical staining, the sections were incubated with primary antibodies overnight at 4 °C, Then incubate with secondary antibodies (or fluorescence-label) at room temperature for 1-1.5 h. Image-J was used for analysis and the scale bar represents 100 μm.

3. Cell immunostaining

At room temperature, Cells were fixed with 4% formaldehyde for 30 min and permeabilized with 0.2% Triton X-100 for 20 min. Then, the cells were incubated with the primary antibodies overnight at 4°C and secondary antibodies for 1 h at room temperature. Then cells were stained with DAPI, images were obtained by confocal microscope (Leica SP8), image-j was used for analysis and and the scale bar represents 100 μm.

4. Western blot analysis

Heart tissues and cultured cells were used radioimmunoprecipitation assay (RIPA) lysis buffer (Beyotime, China) supplemented phosphatase and protease inhibitors (Fdbio, China) to obtain total protein. After gel preparation, gel running, membrane transfer, blocking and primary antibodies incubation at 4°C overnight. The protein bands were incubated with The secondary antibodies included goat anti-rabbit IgG-HRP and goat anti-mouse IgG-HRP (1:5000, Proteintech, USA) and visualized

with ECL substrate (Fdbio, China) by the Gene Gnome Imaging System (Syngene Bio Imaging, USA). Image-J was used for analysis.

5. Chemicals and reagents

Serum Elisa kits were purchased from Jiangsu Jingmei Biological Technology Corporation. Mouse brain natriuretic peptide (BNP) ELISA Kit (JM-02343M1), Mouse Interleukin 6 (IL-6) ELISA Kit (JM-02446M1), Mouse transforming growth factor-β (TGF-β) ELISA Kit (JM-02969M1), Mouse Tumour Necrosis Factor-α (TNF-α) ELISA Kit (JM-02415M1), Mouse Interferon beta (IFN-β) ELISA Kit (JM-02407M1), Mouse total cholesterol (TC) ELISA kit (JM-02912M1), Mouse triglyceride (TG) ELISA kit (JM-02911M1)

6. Cell viability assay

Cell viability was assessed via a Cell Counting Kit-8 (CCK-8) assay (Beyotime, China) according to the manufacturer's protocol. In brief, the cells were inoculated into 96-well cell dishes. After the corresponding cell stimulation, CCK-8 reagent (20 μL) was added to each well and mixed with appropriate volumes of cell culture medium. Wells without cells served as blank controls. After 2 hours of culture at 37°C, the absorbance at 450 nm was measured using a microplate reader.

7. Coimmunoprecipitation

The protein concentrations of the NRCMs and H9C2 cells were determined with a BCA protein assay kit (Thermo Fisher Scientific, USA). Total protein lysates (1 mg) from each sample were used for immunoprecipitation (IP). The lysates were incubated with the IgG control or anti-PINK1 antibody overnight at 4 °C. Subsequently, the

samples were incubated with protein A/G beads (Absin). After being washed and denatured with immunoprecipitation buffer, the eluted proteins were subjected to western blotting and silver staining.

Supplement Figures

Figure S1. PINK1 deficiency aggravates cardiac dysfunction in HFpEF mice. (A) The weight of mice in each group for every 4 weeks. (B) Quantitative analyses of HW/BW (Heart weight normalized to body weight), HW/LW (Heart weight-to-lung weight ratio), systolic blood pressure (SBP), serum glucose (n = 8 in each group). (C) Quantitative analyses of serum cholesterol (TC), triglycerides (TG), BNP, IFN-β, IL-6, TNF-α, TGF-β(n = 5 in each group). For all statistical plots, the data are presented as the means ± SEs. *P<0.05; **P<0.01; ***P<0.001.

Figure S2. PINK1 overexpression attenuates cardiac dysfunction in HFpEF mice. (A) The weight of mice in each group for every 4 weeks. (B) Quantitative analyses of HW/BW (Heart weight normalized to body weight), HW/LW (Heart weight-to-lung weight ratio), systolic blood pressure (SBP), serum glucose (n = 8 in each group). (C) Quantitative analyses of serum cholesterol (TC), triglycerides (TG), BNP, IFN-β, IL-6, TNF-α, TGF-β were measured using multiplex immunoassay (n = 5 in each group). For all statistical plots, the data are presented as the means ± SEs. *P<0.05; **P<0.01; ***P<0.001.

Figure S3. siPINK1 exacerbates palmitic acid-induced apoptosis and decreased mitochondrial function in NRCMs. (A) Fluorescence images of MMP detected by JC-1 tracker. J-aggregate staining is shown in red and J-monomer staining is shown in

green. (B) Apoptotic cardiomyocytes (red) were examined by TUNEL staining. For all statistical plots, the data are presented as the means ± SEs. *P<0.05; **P<0.01; ***P<0.001.

Figure S4. adPINK1 attenuates palmitic acid-induced apoptosis and increased mitochondrial function in NRCMs. (A) Fluorescence images of MMP detected by JC-1 tracker. J-aggregate staining is shown in red and J-monomer staining is shown in green. (B) Apoptotic cardiomyocytes (red) were examined by TUNEL staining. For all statistical plots, the data are presented as the means ± SEs. *P<0.05; **P<0.01; ***P<0.001.

Figure S5. siPrdx2 restored the mitochondrial function of adPINK1 on NRCM lipotoxicity (A) Fluorescence images of MMP detected by JC-1 tracker. J-aggregate staining is shown in red and J-monomer staining is shown in green. For all statistical plots, the data are presented as the means ± SEs. *P<0.05; **P<0.01; ***P<0.001.

Figure S6. (A) PINK1 structure detail in uniprot database. (B) Peptides detected by mass spectrometry of the three groups

Figure S7. The abundance of three groups on the proteins in the biological process, cellular component, and molecular function.

Figure S8: Genomic PCR analysis of mouse tail genomic DNA from PINK1-KO mice.

Figure S9: Generation of cardiac-specific PINK1 overexpression mice. (A) The information of cardiac-specific PINK1 overexpression mouse generation protocol. (B) The information of cardiac-specific PINK1 overexpression mouse identification

protocol. (C) Genomic PCR analysis of mouse tail genomic DNA from PINK1-Tg mice.

Figure S10: mRNA level and Cycloheximide Chase (CHX) Assay of Prdx2 in PINK1 overexpression. (A) Cycloheximide Chase (CHX) Assay of Prdx2 in PINK1 overexpression. (B) Relative mRNA levels of Prdx2 For all statistical plots, the data are presented as the means ± SEs. *P<0.05; **P<0.01; ***P<0.001.

Figure S11: Chloroquine doesn't affect Prdx2 mRNA and protein expresson in PINK1 overexpresses. (A) Western blot for the expression of PINK1, Prdx2, β-actin. (B) Relative mRNA levels of Prdx2. For all statistical plots, the data are presented as the means ± SEs. *P<0.05; **P<0.01; ***P<0.001.

Figure S12: mito-SOX staining and MDA content of NRCMs. (A) mito-SOX staining of NRCMs Scale bar, 50 μm. (B) MDA amount of NRCMs (n = 5 in each group). For all statistical plots, the data are presented as the means ± SEs. *P<0.05; **P<0.01; ***P<0.001.

Figure S13: Western blotting for the expression of Prdx2, Bcl2, Bax, Cleaved-Caspase3 and β-actin in NRCMs. For all statistical plots, the data are presented as the means ± SEs. *P<0.05; **P<0.01; ***P<0.001.

Figure S14: The content of cardiac mtDNA and serum sST2, galectin-3, cTNI, CK-MB, and MPO. (A) Relative mRNA levels of mt-Nd1 and mt-Cytb in mitochondria and cytoplasm. (B) serum content of sST2, galectin-3, cTNI, CK-MB, and MPO. For all statistical plots, the data are presented as the means ± SEs. *P<0.05; **P<0.01; ***P<0.001.

Supplement Table and Date

Supplement 1: raw date about mass spectrometry of the PINK1-targeted protein.

Supplement 2: raw date about Mass spectrometry report of three group (CPF-1 represents PINK1(Full length)-GFP; CPF-2 represents PINK1(1-139aa)-GFP; CPF-3 represents PINK1(140-580aa)-GFP).

Supplement 3: Generation and sequence information of PINK1(Full length)-GFP; PINK1(1-139aa)-GFP; PINK1(140-580aa)-GFP plasmids.

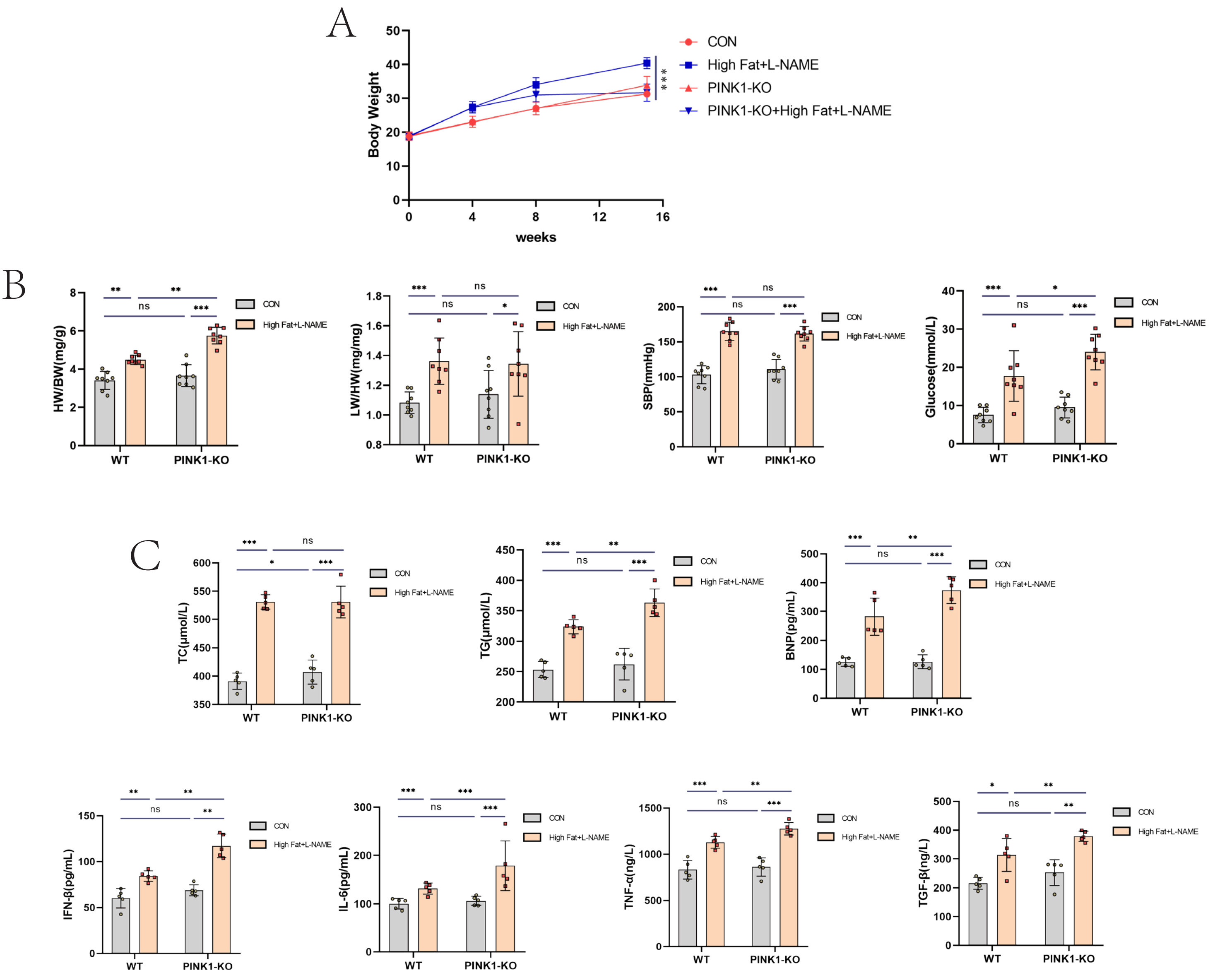

Figure S1. PINK1 deficiency aggravates cardiac dysfunction in HFpEF mice. (A) The weight of mice in each group for every 4 weeks. (B) Quantitative analyses of HW/BW (Heart weight normalized to body weight), HW/LW (Heart weight-to-lung weight ratio), systolic blood pressure (SBP), serum glucose (n = 8 in each group). (C) Quantitative analyses of serum cholesterol (TC), triglycerides (TG), BNP, IFN-β, IL-6, TNF-α, TGF-β (n = 5 in each group). For all statistical plots, the data are presented as the means ± SEs. *P<0.05; **P<0.01; ***P<0.001using Student t test.

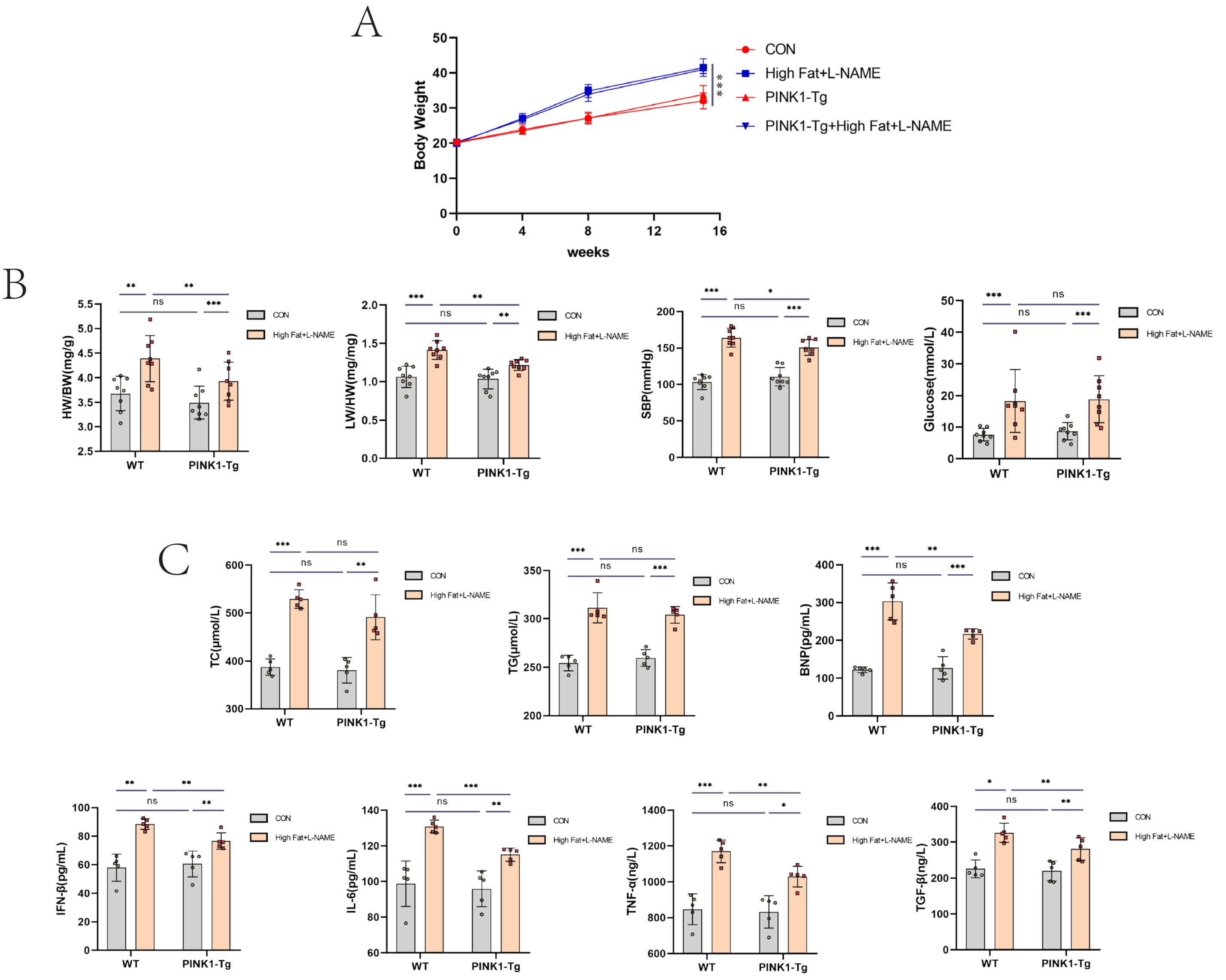

Figure S2. PINK1 overexpression attenuates cardiac dysfunction in HFpEF mice. (A) The weight of mice in each group for every 4 weeks. (B) Quantitative analyses of HW/BW (Heart weight normalized to body weight), HW/LW (Heart weight-to-lung weight ratio), systolic blood pressure (SBP), serum glucose (n = 8 in each group). (C) Quantitative analyses of serum cholesterol (TC), triglycerides (TG), BNP, IFN-β, IL-6, TNF-α, TGF-β were measured using multiplex immunoassay (n = 5 in each group). For all statistical plots, the data are presented as the means ± SEs. *P<0.05; **P<0.01; ***P<0.001 using Student t test.

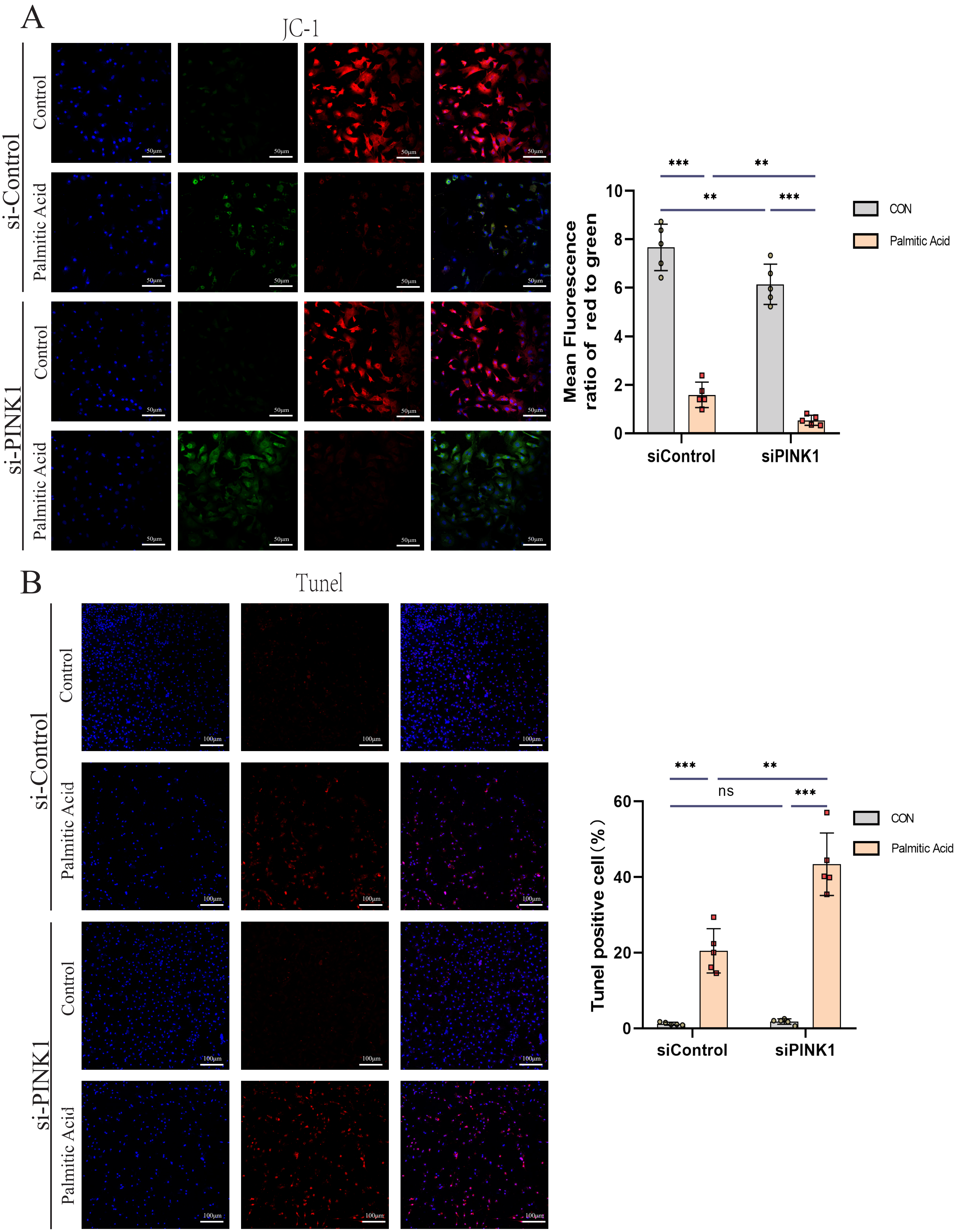

Figure S3. siPINK1 exacerbates palmitic acid-induced apoptosis and decreased mitochondrial function in NRCMs. (A) Fluorescence images of MMP detected by JC-1 tracker. J-aggregate staining is shown in red and J-monomer staining is shown in green. Scale bar, 50 μm. (B) Apoptotic cardiomyocytes (red) were examined by TUNEL staining. Scale bar, 100 μm. For all statistical plots, the data are presented as the means ± SEs. *P<0.05; **P<0.01;

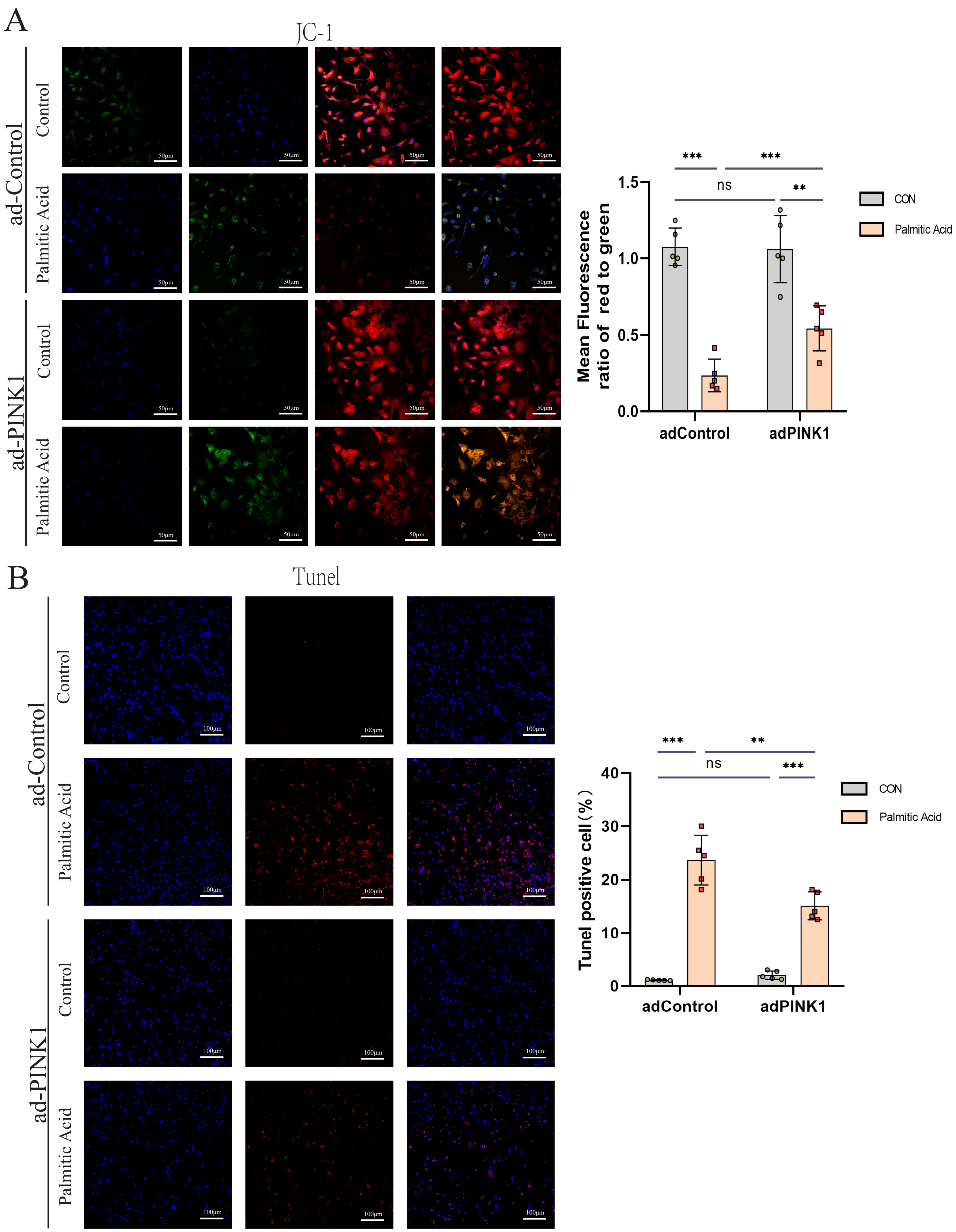

Figure S4. adPINK1 attenuates palmitic acid-induced apoptosis and increased mitochondrial function in NRCMs. (A) Fluorescence images of MMP detected by JC-1 tracker. J-aggregate staining is shown in red and J-monomer staining is shown in green. Scale bar, 50 μm. (B) Apoptotic cardiomyocytes (red) were examined by TUNEL staining. Scale bar, 100 μm. For all statistical plots, the data are presented as the means ± SEs. *P<0.05; **P<0.01;

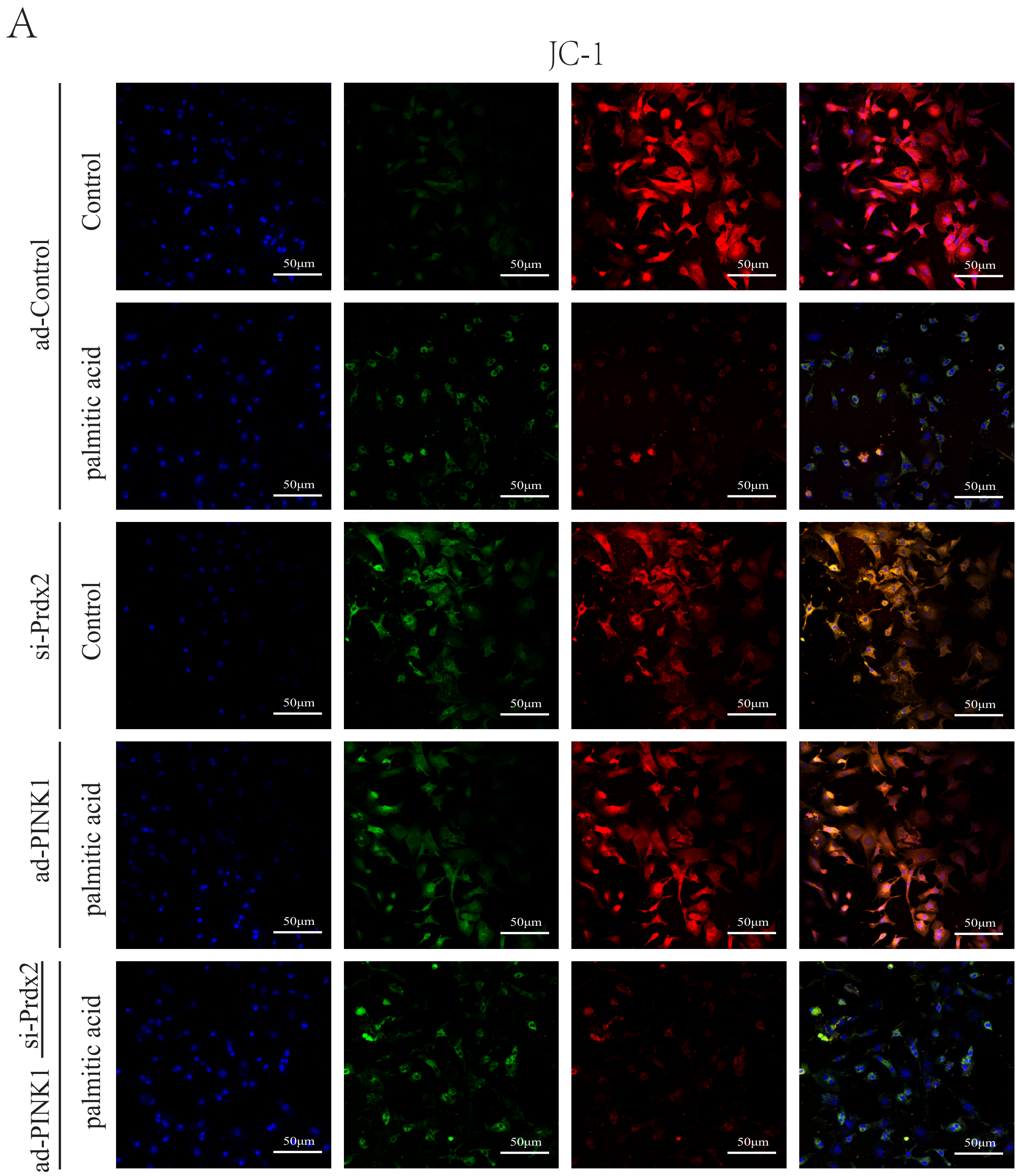

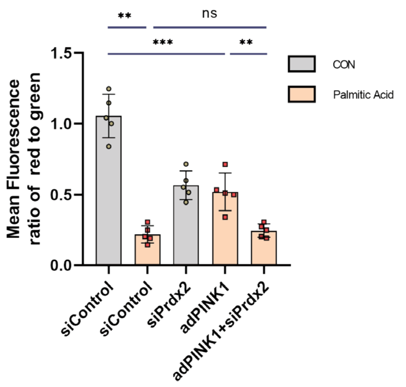

Figure S5. siPrdx2 restored the mitochondrial function of adPINK1 on NRCM lipotoxicity (A) Fluorescence images of MMP detected by JC-1 tracker. J-aggregate staining is shown in red and J-monomer staining is shown in green. Scale bar, 50 μm. For all statistical plots, the data are presented as the means ± SEs. *P<0.05; **P<0.01; ***P<0.001 using Student t test.

A

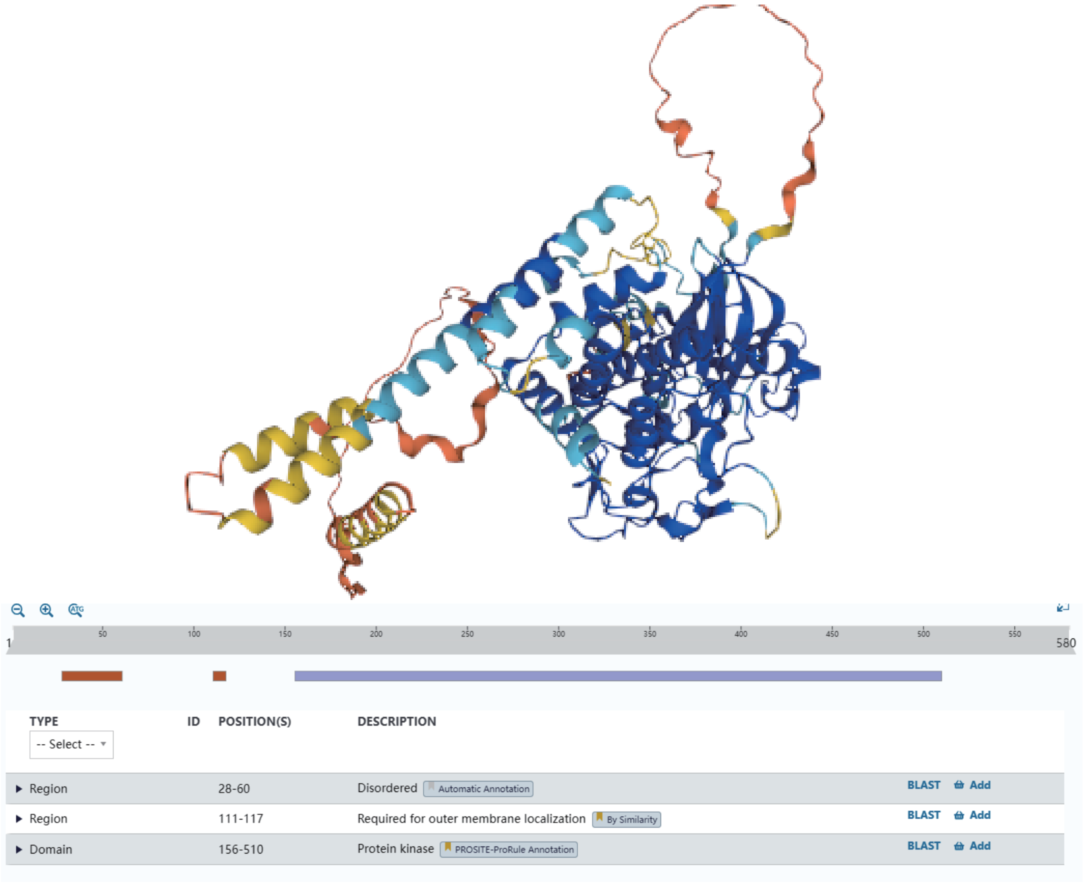

B

MASGNAHIGK PAPDFTGTAV VDGAFKEIKL SDYRGKYVVL FFYPLDFTFV CPTEIIAFSD
HAEDFRKLGC EVLGVSVDSQ FTHLAWINTP RKEGGLGPLN IPLLADVTKS LSQNYGVLKN
DEGIAYRGLF IIDAKGVLRQ ITVNDLPVGR SVDEALRLVQ AFQYTDEHGE VCPAGWKPGS
DTIKPNVDDS KEYFSKHN

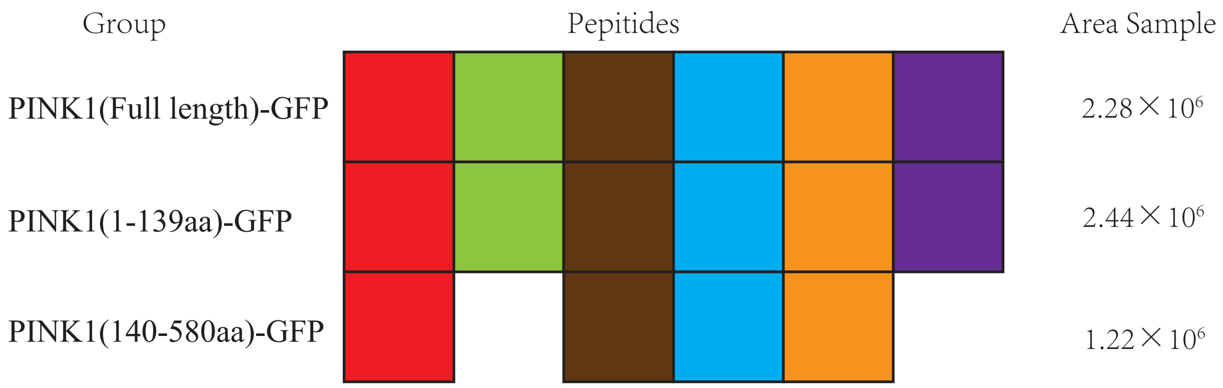

Figure S6. (A) PINK1 structure detail in uniprot database. (B) Peptides detected by mass spectrometry of the three groups

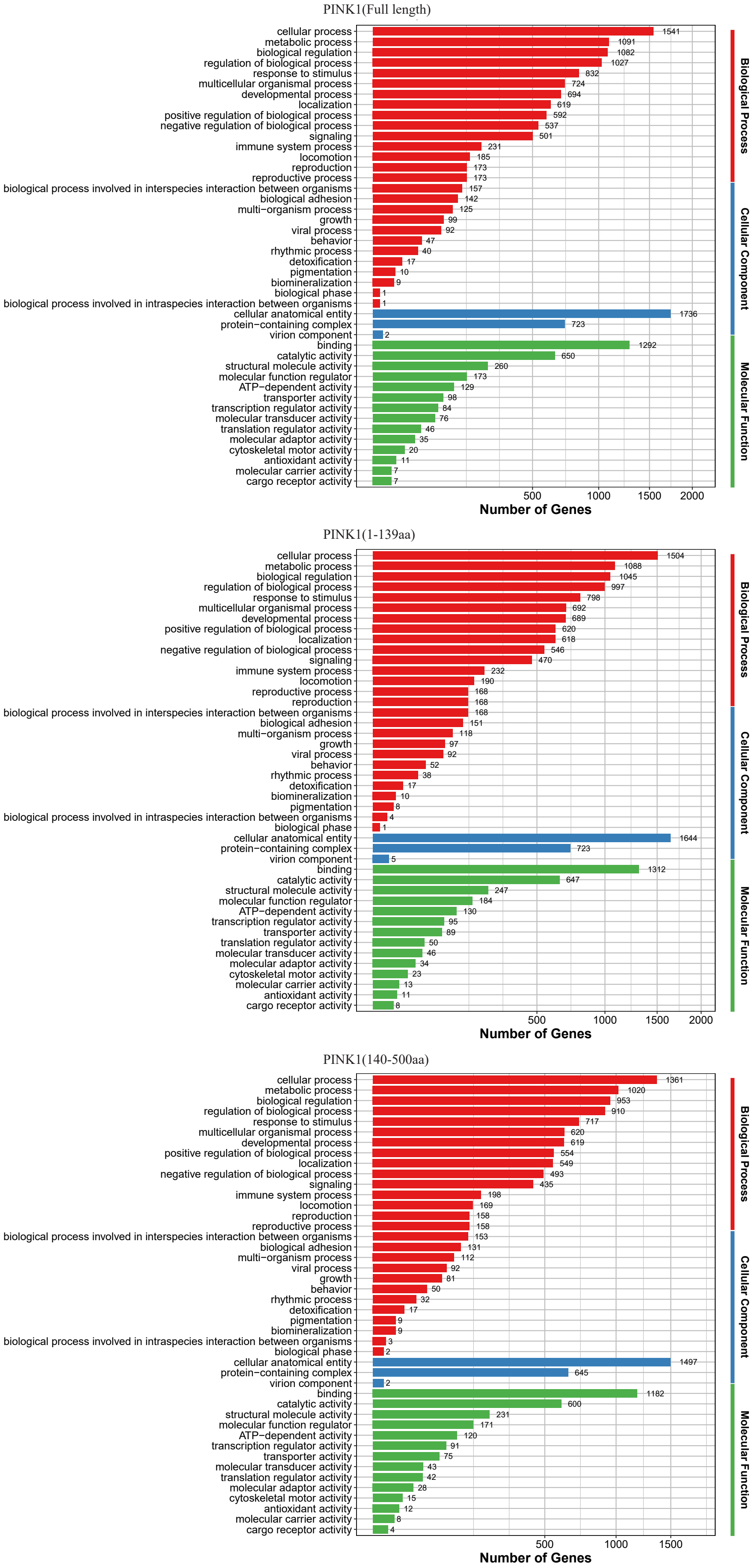

Figure S7. The abundance of three groups on the proteins in the biological process, cellular component, and molecular function.

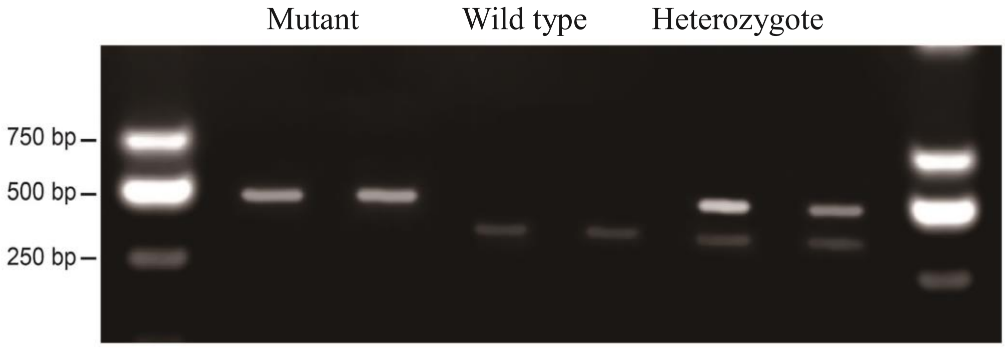

Figure S8: Genomic PCR analysis of mouse tail genomic DNA from PINK1-KO mice.

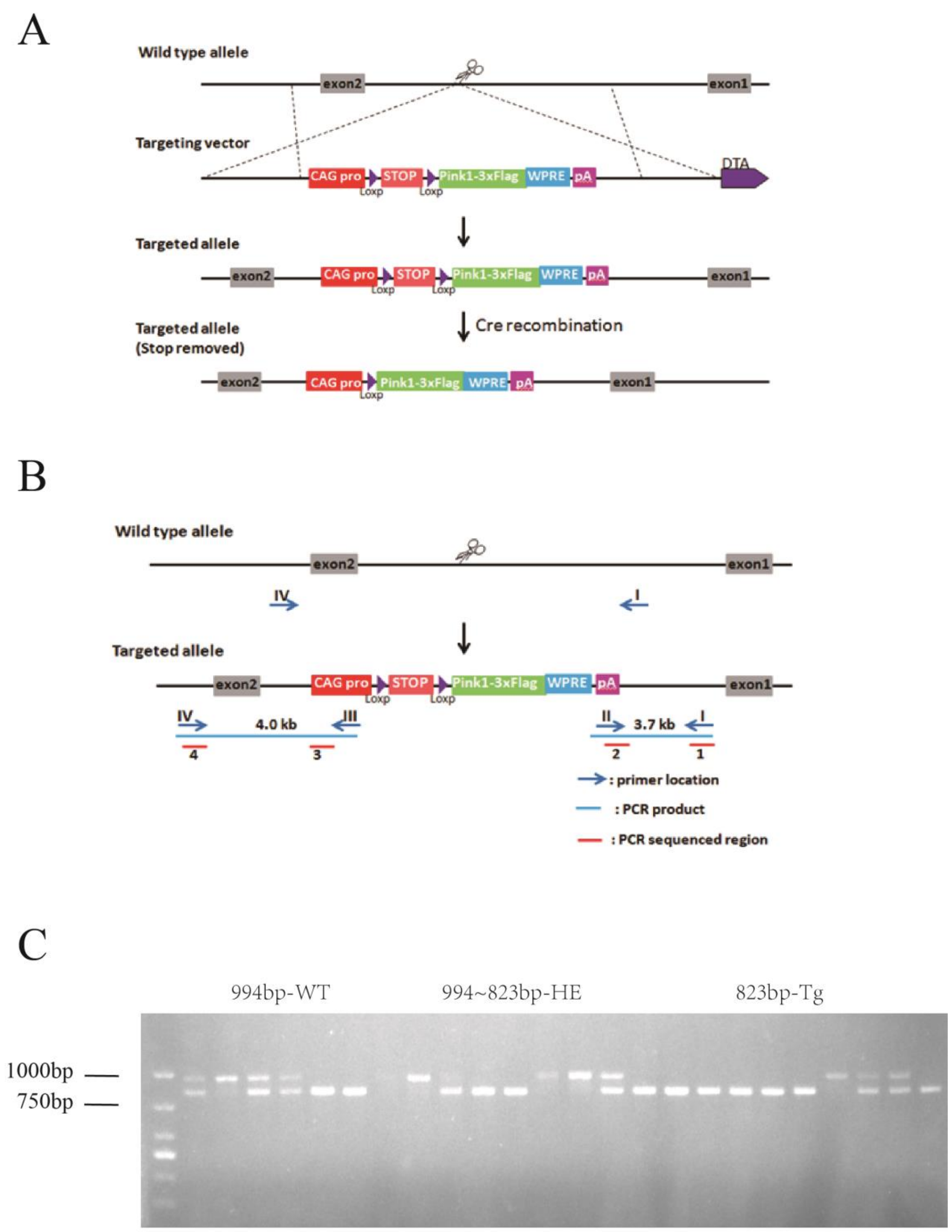

Figure S9: Generation of cardiac-specific PINK1 overexpression mice. (A) The information of cardiac-specific PINK1 overexpression mouse generation protocol. (B) The information of cardiac-specific PINK1 overexpression mouse identification protocol. (C) Genomic PCR analysis of mouse tail genomic DNA from PINK1-Tg mice.

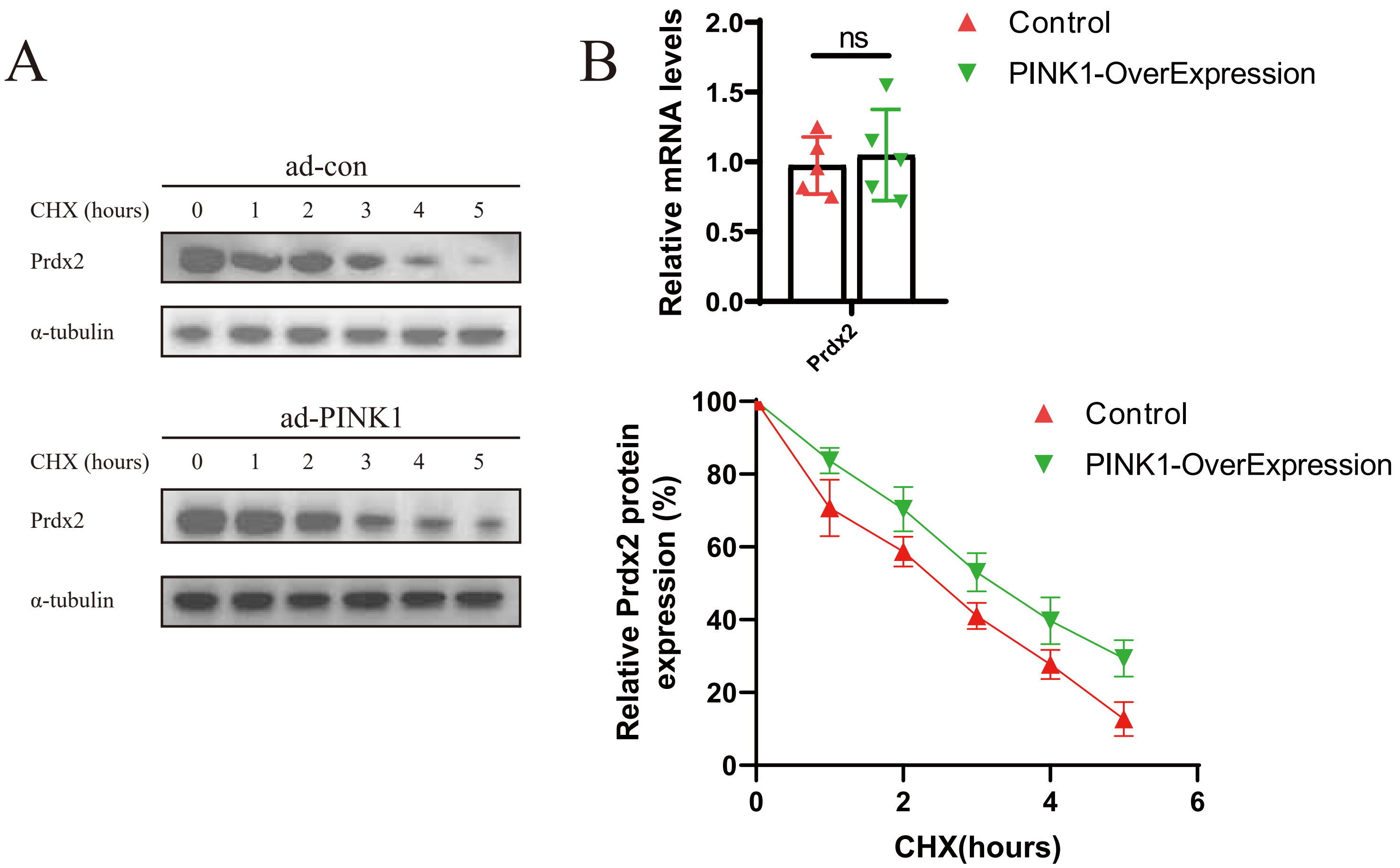

Figure S10: mRNA level and Cycloheximide Chase (CHX) Assay of Prdx2 in PINK1 overexpression. (A) Cycloheximide Chase (CHX) Assay of Prdx2 in PINK1 overexpression. (B) Relative mRNA levels of Prdx2.

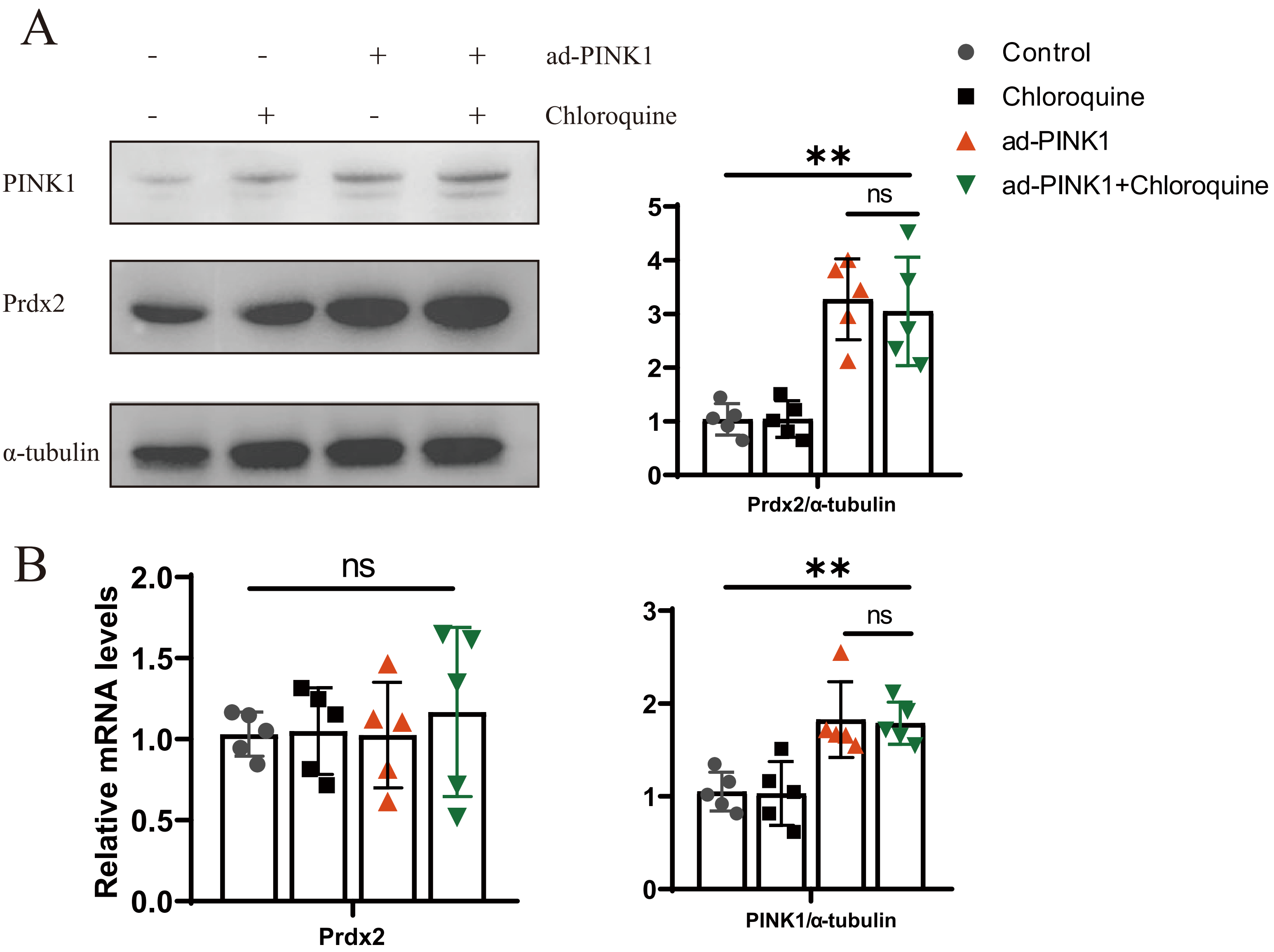

Figure S11: Chloroquine doesn't affect Prdx2 mRNA and protein expresson in PINK1 overexpresses. (A) Western blot for the expression of PINK1, Prdx2, β-actin. (B) Relative mRNA levels of Prdx2.

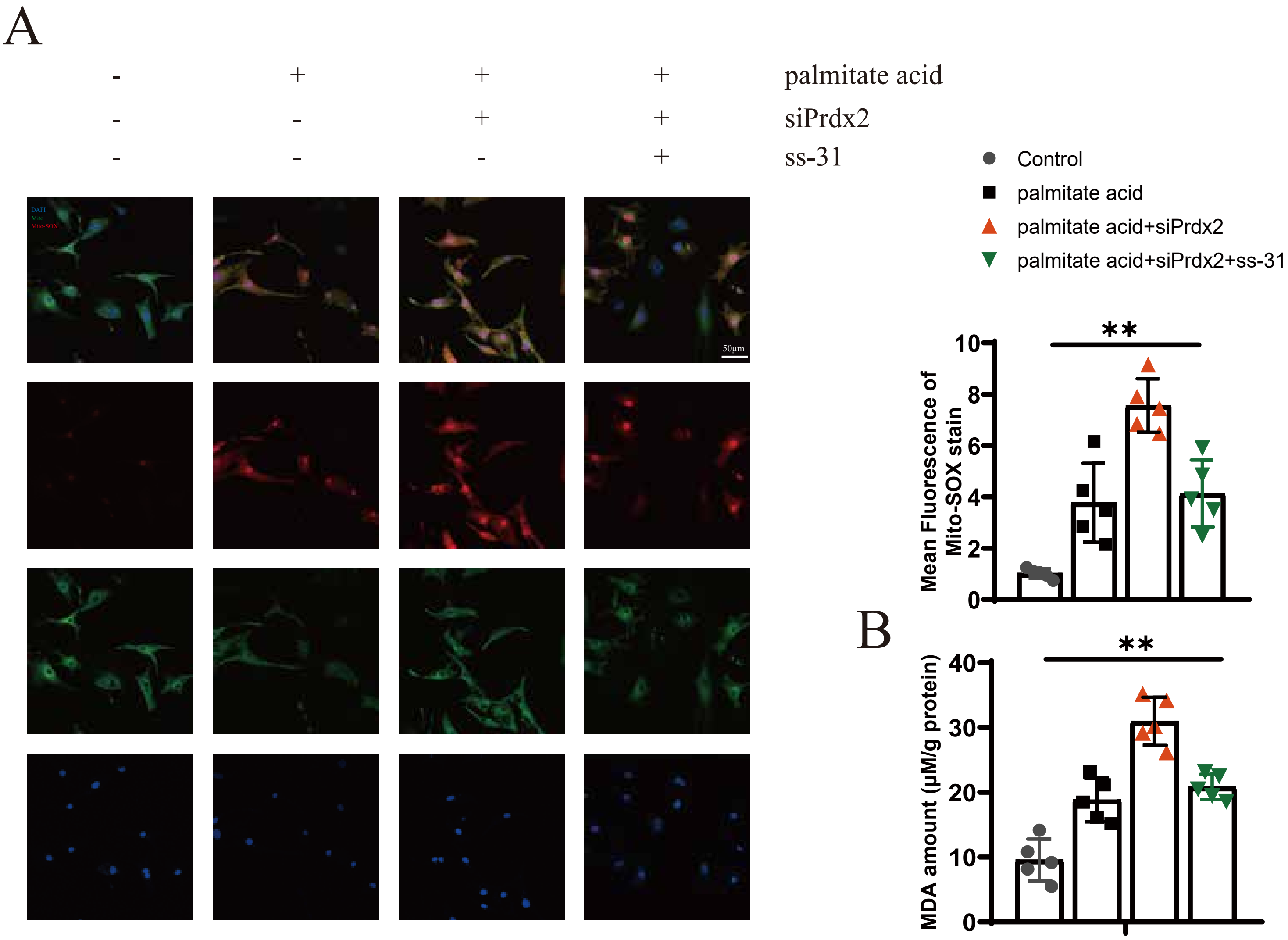

Figure S12: mito-SOX staining and MDA content of NRCMs. (A) mito-SOX staining of NRCMs Scale bar, 50 μm. (B) MDA amount of NRCMs (n = 5 in each group).

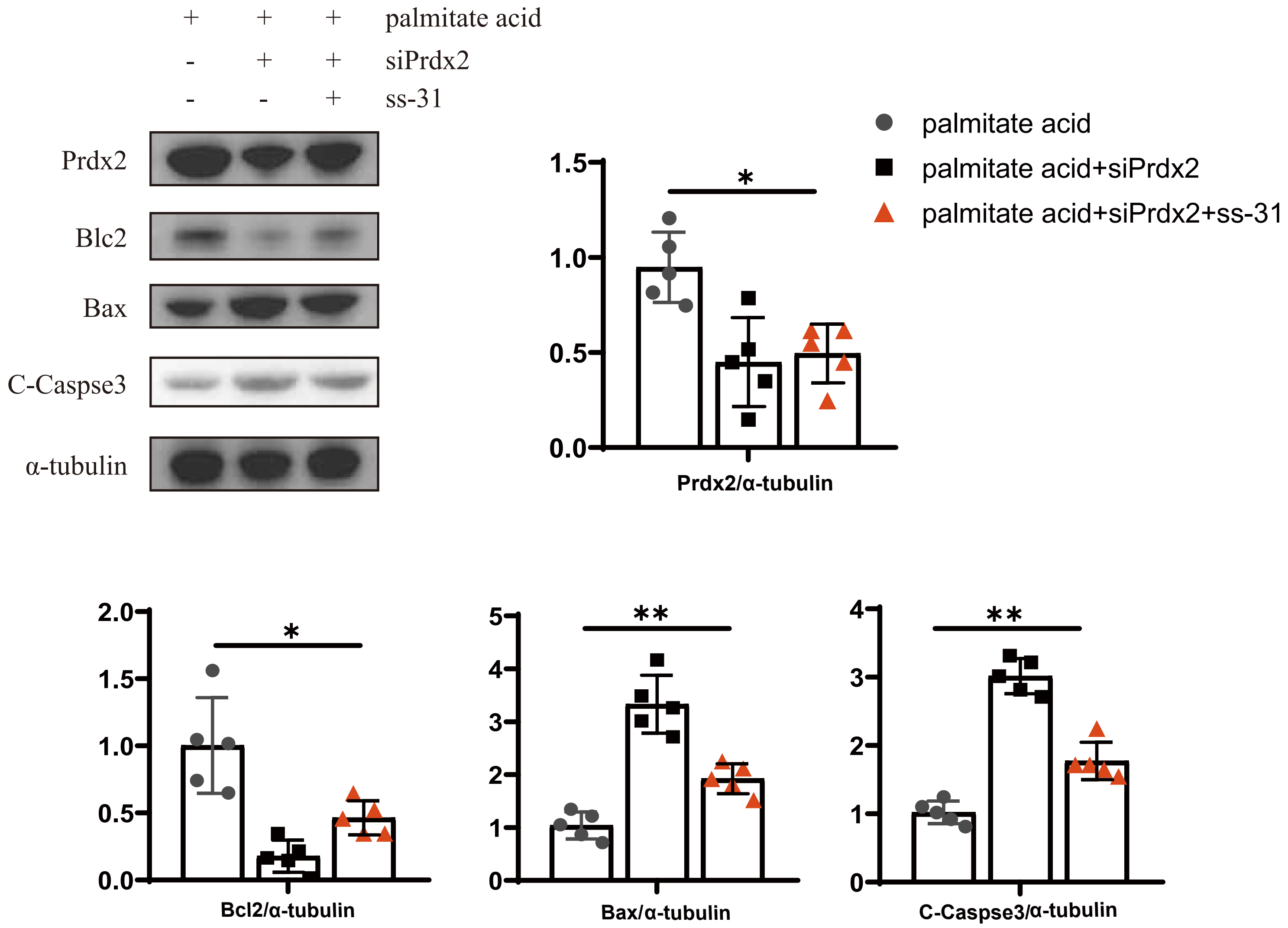

Figure S13: Western blotting for the expression of Prdx2, Bcl2, Bax, Cleaved-Caspase3 and β-actin in NRCMs.

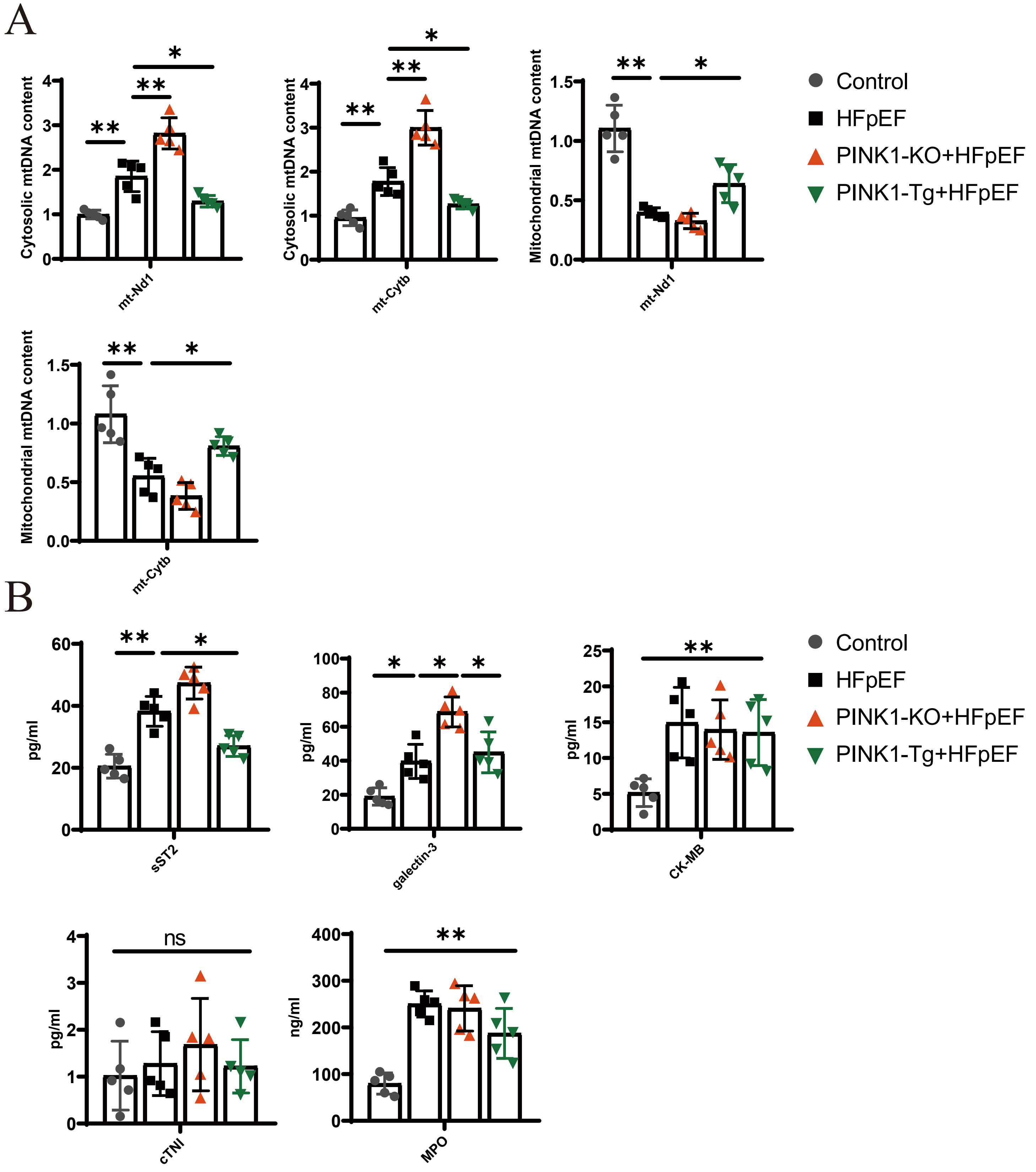

Figure S14: The content of cardiac mtDNA and serum sST2, galectin-3, cTNI, CK-MB, and MPO. (A) Relative mRNA levels of mt-Nd1 and mt-Cytb in mitochondria and cytoplasm. (B) serum content of sST2, galectin-3, cTNI, CK-MB, and MPO.

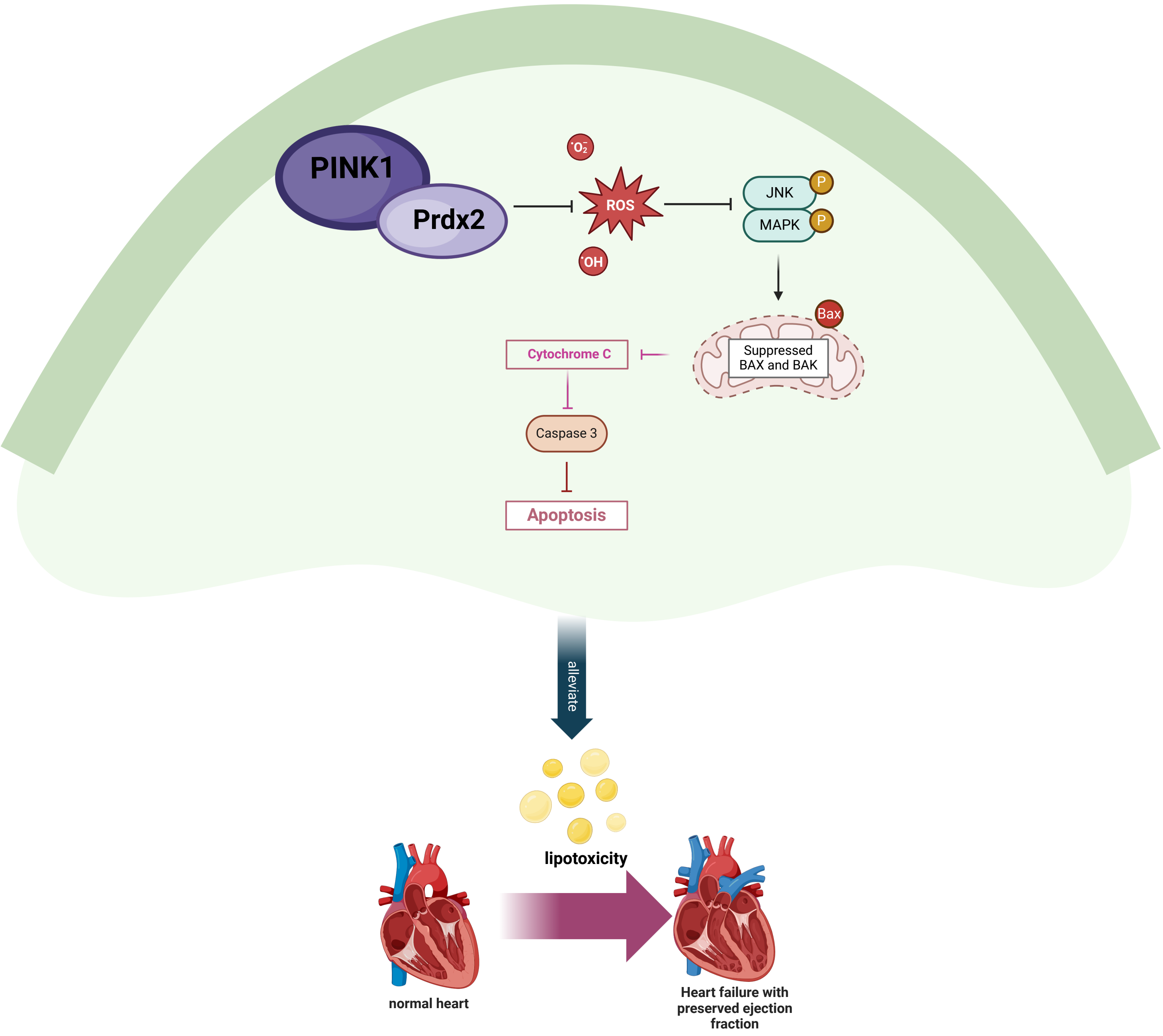
PINK1
Prdx2
ROS
JNK
MAPK
P
P
Bax
Suppressed BAX and BAK
Cytochrome C
Caspase 3
Apoptosis
alleviate
lipotoxicity
normal heart
Heart failure with preserved ejection fraction

Highlights:

Our investigation discloses a pivotal relationship between PINK1 and Prdx2 in the context of HFpEF.

Notably, PINK1, in addition to its role in mitochondrial autophagy, can increase Prdx2 expression, effectively remove ROS and attenuate cardiomyocyte apoptosis by modulating the JNK and p38 pathways, thereby alleviating myocardial lipotoxicity and improving HFpEF cardiac function.

Our studies offer valuable insights, opening avenues for the development of innovative therapeutic strategies in the prevention and treatment of HFpEF.
